# Phosphodiesterase 4D promotes angiotensin II-induced hypertension in mice via smooth muscle cell contraction

**DOI:** 10.1038/s42003-022-03029-0

**Published:** 2022-01-20

**Authors:** Tianfei Fan, Yangfeng Hou, Weipeng Ge, Tianhui Fan, Xiaohang Feng, Wenjun Guo, Xiaomin Song, Ran Gao, Jing Wang

**Affiliations:** State Key Laboratory of Medical Molecular Biology, Institute of Basic Medical Sciences, Chinese Academy of Medical Sciences, Department of Pathophysiology, School of Basic Medicine, Peking Union Medical College, 100005 Beijing, China

**Keywords:** Hypertension, Molecular biology

## Abstract

Hypertension is a common chronic disease, which leads to cardio-cerebrovascular diseases, and its prevalence is increasing. The cyclic adenosine monophosphate (cAMP)-protein kinase A (PKA) pathway participates in multiple cardiovascular diseases. Phosphodiesterase (PDE) 4 has been shown to regulate PKA activity via cAMP specific hydrolysis. However, whether PDE4-cAMP-PKA pathway influences hypertension remains unknown. Herein, we reveal that PDE4D (one of PDE4 isoforms) expression is upregulated in the aortas of experimental hypertension induced by angiotensin II (Ang II). Furthermore, knockout of *Pde4d* in mouse smooth muscle cells (SMCs) attenuates Ang II-induced hypertension, arterial wall media thickening, vascular fibrosis and vasocontraction. Additionally, we find that PDE4D deficiency activates PKA-AMP-activated protein kinase (AMPK) signaling pathway to inhibit myosin phosphatase targeting subunit 1 (MYPT1)-myosin light chain (MLC) phosphorylation, relieving Ang II-induced SMC contraction in vitro and in vivo. Our results also indicate that rolipram, a PDE4 inhibitor, may be a potential drug for hypertension therapy.

## Abstract (web summary)

Fan, Hou et al. investigate the role of phosphodiesterase (PDE) 4 and the PDE4-cAMP-PKA pathway on hypertension, reporting that PDE4D is upregulated in hypertension induced by angiotensin II. The authors also show that Pde4d knockout in mouse SMCs attenuates Ang II-induced hypertension and implicate AMPK signalling and MYPT1-MLC phosphorylation in Ang II-induced SMC contraction.

## Introduction

Hypertension is defined as an arterial systolic and diastolic blood pressure (BP) >140/90 mmHg by European Society of Cardiology/European Society of Hypertension^1^. While generally asymptomatic, hypertension is a severe risk factor for cardiovascular diseases, strokes, and kidney diseases^2^. Hypertension occurs through multiple pathogeneses, including sympathetic activation^3^, the renin-angiotensin-aldosterone system disorder^4^, inflammation^5^, and endothelial cell (EC) and smooth muscle cell (SMC) dysfunction^6,7^. Presently, most hypertension medicines have adverse effects—headaches, oedema, and hyperkalaemia—which limit their application and lead to reduced patient compliance^8^. Besides, there are some hypertension patients who are insensitive to existing antihypertensive drugs, ultimately lead to resistant hypertension^9^. It is therefore imperative to develop potential hypertension treatments.

Phosphodiesterase (PDE), consisting of 11 subfamilies (PDE1–PDE11), is the hydrolase of cyclic adenosine monophosphate (cAMP) and cyclic guanosine monophosphate. Then, PDE4, consisting of four isoforms (PDE4A-D), are cAMP specific hydrolases^10^. PDE4 participates in a variety pathophysiological processes^11^, promoting SMCs’ phenotypic switch and neointima formation in atherosclerosis^12^, as well as aggravating pulmonary arterial hypertension through the regulation of vascular tone and inflammatory factors^13^. In addition, PDE4 inhibitors is an effective treatment strategy for a variety of diseases, including asthma, chronic obstructive pulmonary disease, and psoriasis^14^. Exploring the role of PDE4 isoenzymes in hypertension is vital to the development of new treatment strategies.

As a second messenger, cAMP is related to cardiovascular diseases: cardiac fibrosis, abdominal aortic aneurysm, atherosclerosis, and pulmonary arterial hypertension^15–18^. In addition, the association between cAMP and these diseases was mainly established through its effector protein kinase A (PKA)^17^. However, the roles of PKA and its regulator PDE in hypertension remain unknown. Furthermore, PKA has been shown to activate AMPK^19^. AMPK inhibitor aggravated SMCs contraction and hypertension by activating MYPT1-MLC signaling pathway^20^. As known, one of the pathological processes of hypertension is vasoconstriction, and MYPT1-MLC is a classical cell contraction signaling pathway^21^. Therefore, it is hypothesized that PDE4 may affect SMCs contraction by PKA-AMPK-MYPT1-MLC pathway and thus affect hypertension.

In this study, we found that PDE4D expression was upregulated in aortic tissues of hypertensive mice. Furthermore, PDE4D, expressed in SMCs instead of ECs, contributed to hypertension development. PDE4D deficiency in SMCs and PDE4 inhibitor rolipram reduced Ang II-induced hypertension, and the protective effect of rolipram on hypertension was mainly through PDE4D in SMCs. In addition, we demonstrated that PDE4D promoted SMCs contraction and vasocontraction via PKA-AMPK-MYPT1-MLC signaling pathway.

## Results

### Phosphodiesterase 4D (PDE4D) expression is upregulated in angiotensin (Ang) II-induced hypertensive mice

**Fig. 1:**
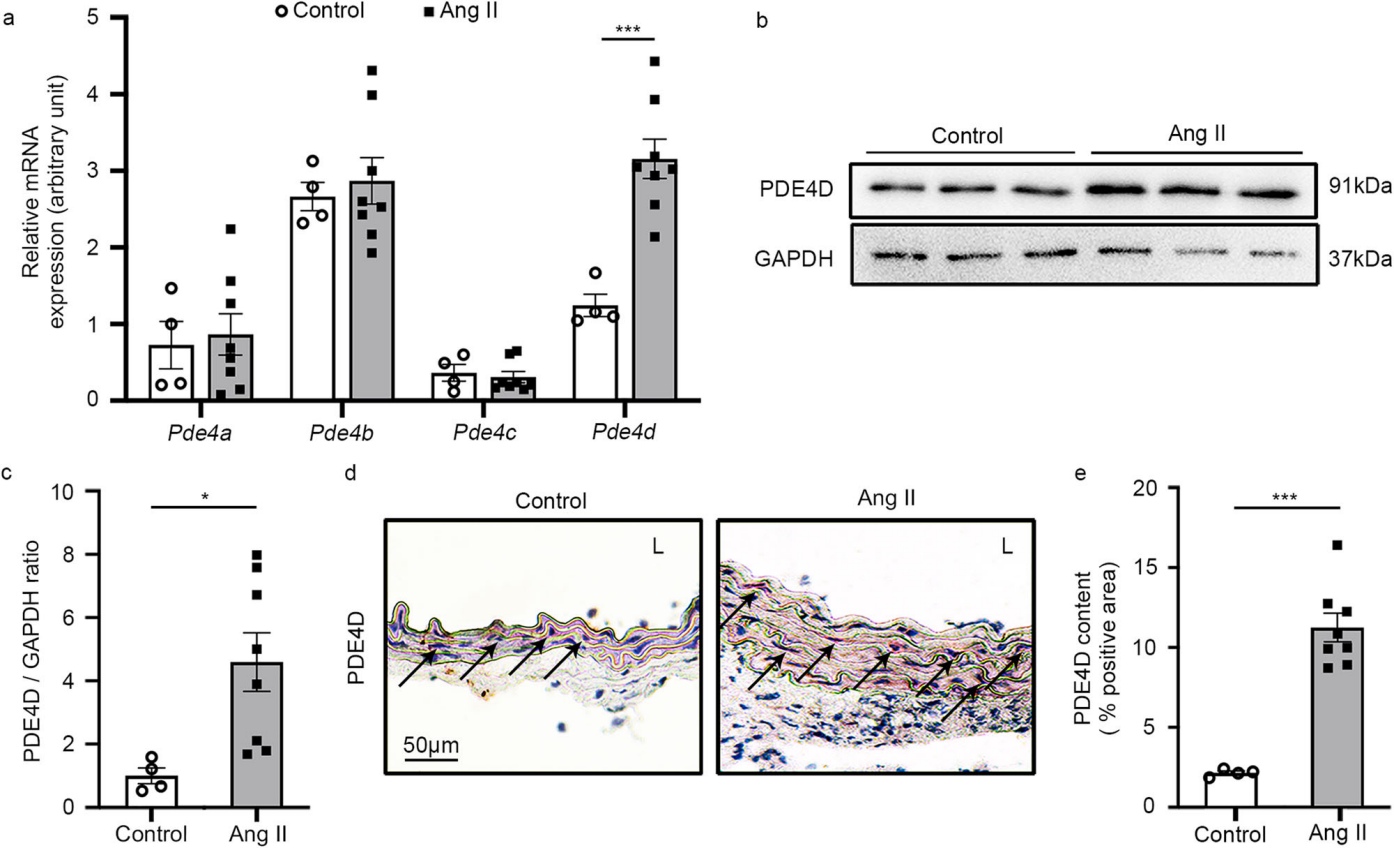
PDE4D expression is upregulated in hypertensive mice. **a** Real-time polymerase chain reaction (RT-PCR) was used to measure mRNA expression of PDE4A, PDE4B, PDE4C, and PDE4D in the aorta tissues of control mice and hypertensive mice (fold change vs. one of controls). **b** Representative western blot showing PDE4D expression in aorta tissues. **c** Quantification of PDE4D expression normalized to glyceraldehyde-3-phosphate dehydrogenase (GAPDH) protein. **d** Representative immunohistochemical staining of PDE4D. Arrows indicate positive areas. **e** Quantification of the percentage of PDE4D-positive area. *n* = 4 in control group, *n* = 8 in hypertensive group. Data are expressed as mean ± standard error of mean (SEM). Two-tailed Student’s *t* test was performed to compare differences between two groups. *p < 0.05, ***p < 0.001. L lumen.

We first established a hypertensive model in wild-type (WT, C57BL/6J) male mice (Supplementary Fig. 1a, b). To initially investigate PDE4 expression after hypertension, we evaluated mRNA levels of each PDE4 isoform (*Pde4a-d*) in control and hypertensive mice aortas. The results revealed a increase in *Pde4d* mRNA level of hypertensive mice (Fig. 1a). The western blot and immunohistochemical staining tests showed that PDE4D expression was increased in hypertensive mice (Fig. 1b–e). However, there was no change in other PDE4 isoforms (Fig. 1a and Supplementary Fig. 2a, b). Together, these findings indicate that PDE4D expression is elevated in Ang II-induced hypertensive mice aortas.

### PDE4D in smooth muscle cells (SMCs) contributes to Ang II-induced mice hypertension

Among vascular intrinsic cell types, EC and SMC are known to play crucial roles in hypertension development^6,7^. To explore whether PDE4D, via SMCs or ECs, plays a role in hypertension, we generated *Pde4d* SMC-specific knockout mice (*Tagln*-Cre mice; *Pde4d*^*SMC−/−*^) and *Pde4d* EC-specific knockout mice (*Tek*-Cre mice; *Pde4d*^*EC−/−*^) mice via Cre-LoxP recombinase system, and confirmed the mice genotypes by agarose gel electrophoresis genotyping and western blot (Supplementary Fig. 3a–e).

**Fig. 2:**
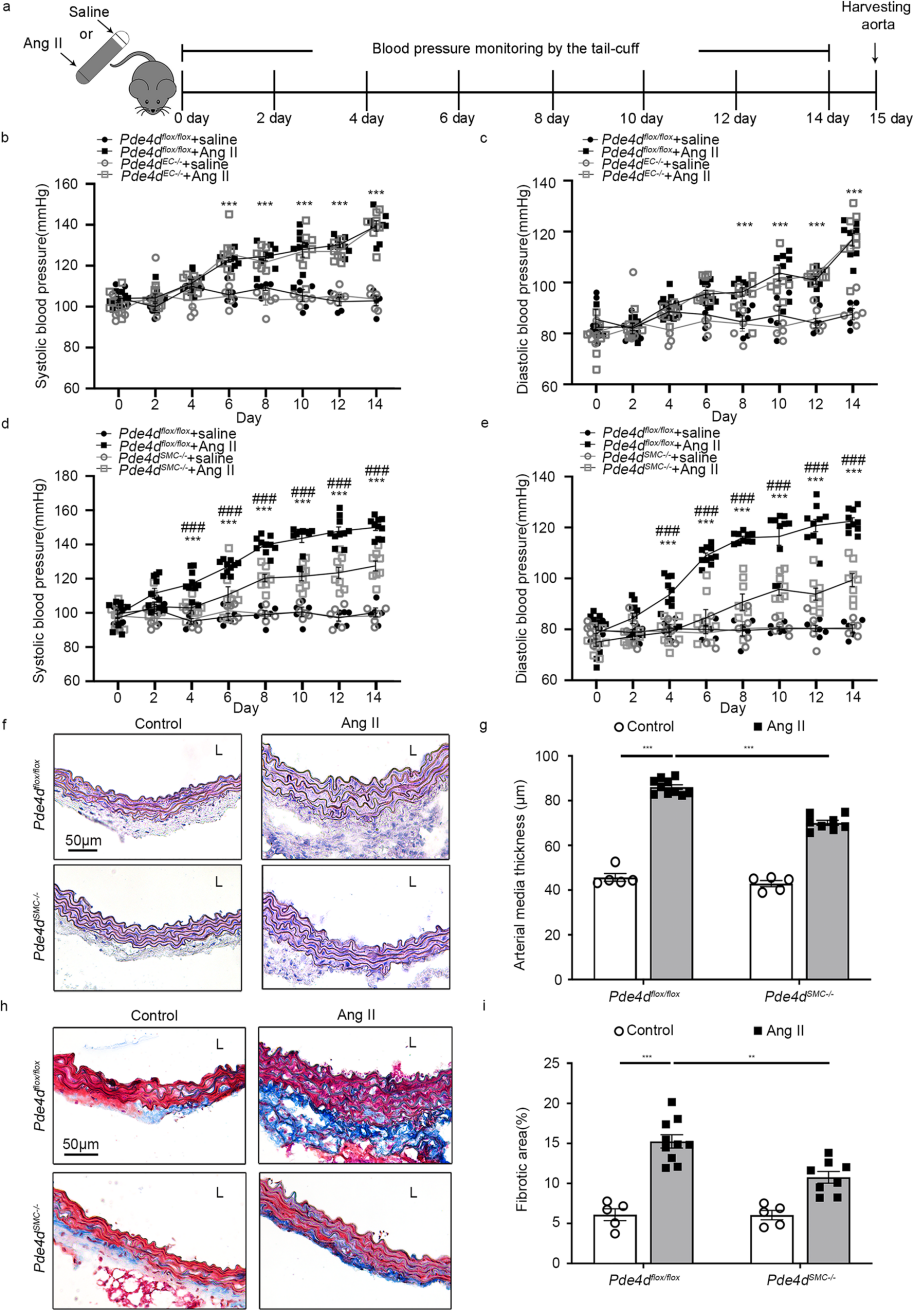
PDE4D in SMCs contributes to Ang II-induced mouse hypertension. **a** Scheme of hypertensive mice inducement. **b** SBP and **c** DBP were measured in *Pde4d*^*flox/flox*^ and *Pde4d*^*EC−/−*^ mice, with or without Ang II treatment: *n* = 5 in *Pde4d*^*flox/flox*^ + saline group, *n* = 8 in *Pde4d*^*flox/flox*^ + Ang II group, *n* = 5 in *Pde4d*^*EC−/−*^ + saline group, and *n* = 8 in *Pde4d*^*EC−/−*^ + Ang II group. **d** SBP and **e** DBP were measured in *Pde4d*^*flox/flox*^ and *Pde4d*^*SMC−/−*^ mice, with or without Ang II treatment: *n* = 5 in *Pde4d*^*flox/flox*^ + saline group, *n* = 10 in *Pde4d*^*flox/flox*^ + Ang II group, *n* = 5 in *Pde4d*^*SMC−/−*^ + saline group, and *n* = 8 in *Pde4d*^*SMC−/−*^ + Ang II group. Data are expressed as mean ± SEM. Two-way ANOVA with Bonferroni’s post hoc test was performed to compare the difference between the multiple groups. ***p < 0.001 for *Pde4d*^*flox/flox*^ + Ang II group vs. *Pde4d*^*flox/flox*^ + saline group, and ^###^*p* < 0.001 for *Pde4d*^*SMC−/−*^ + Ang II group vs. *Pde4d*^*flox/flox*^ + Ang II group. **f** Representative H&E staining under the indicated experimental conditions. **g** Measurement of arterial wall media thickness including all mice in **f**. **h** Representative masson-trichrome staining under the indicated experimental conditions. **i** Quantification of the positively stained area to the aortic wall area including all mice in **h**. Data are expressed as mean ± SEM. Two-way ANOVA with Bonferroni’s post hoc test was performed to compare the difference between the multiple groups. **p < 0.01, ***p < 0.001. L lumen.

We then induced hypertension in two knockout mice groups. Using the tail-cuff method, we measured BP on the first day and every other day during Ang II infusion. After 2 weeks, we harvested the aorta tissues (Fig. 2a). Notably, EC *Pde4d* deficiency (*Pde4d*^*flox/flox*^ + Ang II group vs. *Pde4d*^*EC−/−*^ + Ang II group) did not affect the occurrence or development of the Ang II-induced systolic blood pressure (SBP; 139.3 ± 2.47 mmHg vs. 139.45 ± 2.56 mmHg) or diastolic blood pressure (DBP; 116.85 ± 2.47 mmHg vs. 117.1 ± 3.73 mmHg; Fig. 2b, c). However, SMC *Pde4d* deficiency (*Pde4d*^*flox/flox*^ + Ang II group vs. *Pde4d*^*SMC−/−*^ + Ang II group) inhibited the Ang II-induced increase of both SBP (149.98 ± 1.78 mmHg vs. 127.43 ± 2.97 mmHg, 15.04% reduced by *Pde4d*^*SMC−/−*^ + Ang II group) and DBP (122.48 ± 1.35 mmHg vs. 99.4 ± 2.55 mmHg, 18.84% reduced by *Pde4d*^*SMC−/−*^ + Ang II group; Fig. 2d, e). Hematoxylin and eosin (H&E) staining revealed that Ang II-induced vessel wall media thickening, which was reduced in *Pde4d*^*SMC−/−*^ Ang II infused mice (Fig. 2f, g). In addition, masson-trichrome staining demonstrated that SMC *Pde4d* deficiency reversed Ang II-induced vascular fibrosis (Fig. 2h, i). These results indicate that PDE4D in SMCs, but not in ECs, contribute to Ang II-induced mice hypertension.

### SMC *Pde4d* deficiency reduces vasocontraction

**Fig. 3:**
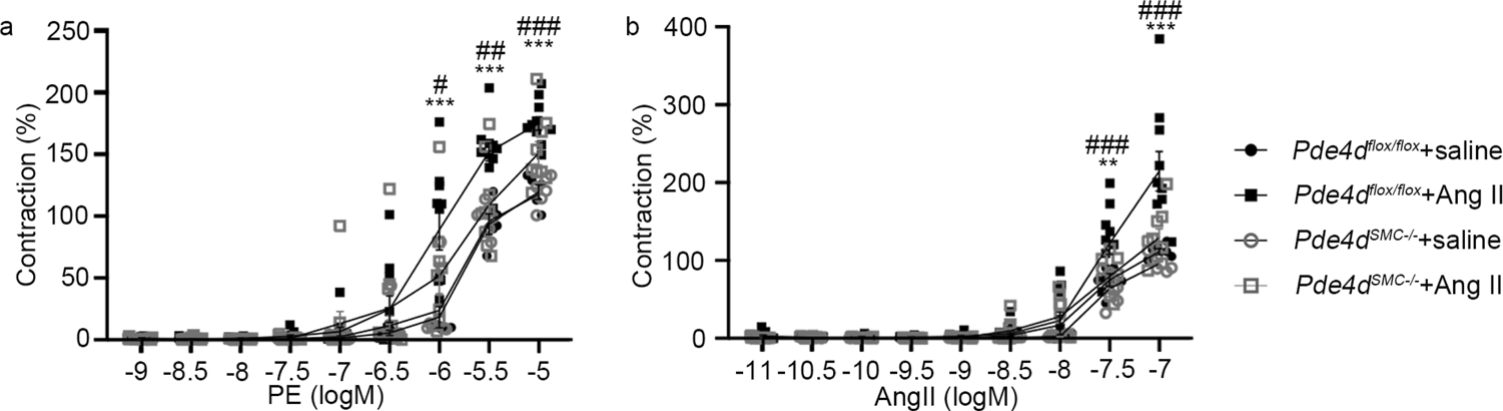
Pde4d deficiency in SMCs affects vasocontraction. Concentration-response curves for **a** PE and **b** Ang II induced vasocontraction of mesenteric resistance artery from *Pde4d*^*flox/flox*^ and *Pde4d*^*SMC−/–*^ mice with or without Ang II treatment (*n* = 5 in *Pde4d*^*flox/flox*^ + saline group, *n* = 10 in *Pde4d*^*flox/flox*^ + Ang II group, *n* = 5 in *Pde4d*^*SMC−/−*^ + saline group, and *n* = 8 in *Pde4d*^*SMC−/−*^ + Ang II group). Data are expressed as mean ± SEM. Two-way ANOVA with Bonferroni’s post hoc test was performed to compare the difference between the multiple groups. **p < 0.01 and ***p < 0.001 for *Pde4d*^*flox/flox*^ + Ang II group vs. *Pde4d*^*flox/flox*^ + saline group. ^#^*p* < 0.05, ^##^*p* < 0.01, and ^###^*p* < 0.001 for *Pde4d*^*SMC−/−*^ + Ang II group vs. *Pde4d*^*flox/flox*^ + Ang II group.

To further explore how PDE4D in SMCs influences hypertension, we examined direct vascular function in the knockout mice. Specifically, the ex vivo vascular function of mesenteric arterioles from *Pde4d*^*flox/flox*^ and *Pde4d*^*SMC−/−*^ mice with or without Ang II treatment. The vasoconstriction of mesenteric arterioles, known as precapillary resistance vessels, causes vascular remodeling and hypertension^22^. Using phenylephrine (PE) and Ang II to induce mesenteric arterioles contraction, we found that the vasocontraction was markedly suppressed in *Pde4d*^*SMC−/−*^ mice compared with *Pde4d*^*flox/flox*^ mice (*Pde4d*^*flox/flox*^ + Ang II group vs. *Pde4*d^*SMC−/−*^ + Ang II group; 10^−5^ M PE: 175.53% ± 5.52% vs. 130.42% ± 10.79%, 25.7% reduced by *Pde4d*^*SMC−/−*^ + Ang II group; 10^−7^ M Ang II: 214.38% ± 25.3% vs. 132.08% ± 12.6%, 38.39% reduced by *Pde4d*^*SMC−/−*^ + Ang II group; Fig. 3a, b). These findings further support that SMC *Pde4d* deficiency relieves vasocontraction.

### PDE4D promotes SMCs contraction via the PKA-AMPK-MYPT1-MLC signaling pathway in vitro

**Fig. 4:**
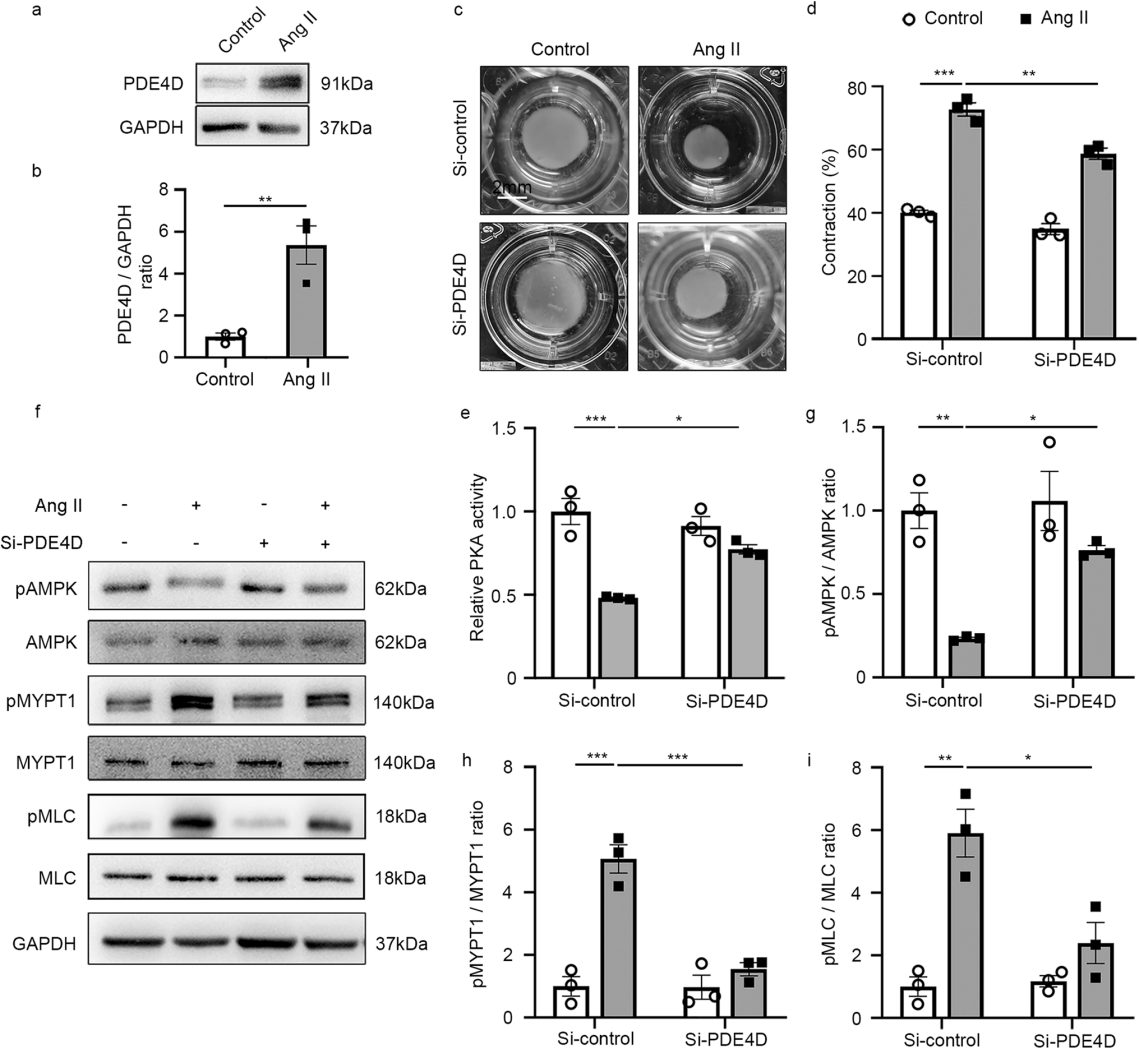
PDE4D promotes SMC contraction through the PKA-AMPK-MYPT1-MLC signaling pathway in vitro. **a** Representative western blot showing PDE4D expression in control and Ang II-stimulated RASMCs. **b** Quantification of PDE4D expression normalized to GAPDH protein. **c** Representative images showing RASMCs contraction. **d** Quantification of RASMCs contraction. **e** Protein kinase A (PKA) kinase activity in RASMCs. **f** Representative western blot exhibiting phosphor-AMPK (pAMPK), AMPK, phospho-MYPT1 (pMYPT1), MYPT1, phospho-MLC (pMLC), and MLC expression in RASMCs. **g** Quantification of pAMPK expression normalized to AMPK protein. **h** Quantification of pMYPT1 expression normalized to MYPT1 protein. **i** Quantification of pMLC expression normalized to MLC protein. *n* = 3 per group. Data are expressed as mean ± SEM. Two-tailed Student’s *t* test was performed to compare differences between two groups and two-way ANOVA with Bonferroni’s post hoc test was performed to compare the difference between the multiple groups. *p < 0.05, **p < 0.01, and ***p < 0.001.

To determine PDE4D’s role in regulating SMC contraction, we evaluated its impact on rat aorta smooth muscle cells (RASMCs) in vitro. First, we verified that PDE4D protein expression but not PDE4A-C was elevated by 5.37-fold in RASMCs after Ang II stimulation (100 nM, 24 h; Fig. 4a, b and Supplementary Fig. 4a, b). Consistently, we found that it was *Pde4d* upregulation instead of other PDE4 isoforms in RASMCs treated with Ang II in mRNA level (Supplementary Fig. 4c). Immunofluorescence staining also showed that PDE4D was increased by Ang II in vitro (Supplementary Fig. 4d). Besides, PDE4 activity was increased by 2.34-fold with Ang II stimulation (Supplementary Fig. 4e). These results indicated that PDE4D was upregulated by Ang II in vitro. Then, we introduced PDE4D small-interfering RNA (siRNA) to validate whether Ang II induces SMCs contraction via PDE4D. After PDE4D siRNA administration, only PDE4D expression, instead of other PDE4 isoforms, was reduced in RASMCs’ mRNA and protein levels (Supplementary Fig. 5a–c). PDE4D was reduced by PDE4D siRNA through immunofluorescence staining (Supplementary Fig. 5d). Indeed, PDE4 activity was also suppressed when PDE4D was knockdown (Supplementary Fig. 5e). We next performed a collagen gel cell contraction assay to explore RASMCs contraction, which revealed that while Ang II promoted RASMC contraction, the addition of PDE4D siRNA inhibited it (si-control + Ang II vs. si-PDE4D + Ang II: 72.68% ± 2.11% vs. 58.65% ± 1.76%) (Fig. 4c, d).

PDE4 family specifically hydrolyzes cAMP to inhibit PKA activity^23–25^. PKA phosphorylates AMPKα at Thr-172 through LKB1 signaling, ultimately leading to AMPK activation^19,26–30^. As such, we investigated whether PDE4D inhibited PKA and AMPK activity in RASMCs. Consistently, we found that Ang II stimulation reduced PKA activity in RASMCs, and that PDE4D siRNA reversed this effect (Fig. 4e). Moreover, we observed that PDE4D siRNA increased Ang II-reduced AMPK phosphorylation (Fig. 4f, g). Meanwhile, we used PKA inhibitor (PKI, 10 μM, 60 min) to further confirm whether PDE4D regulated pAMPK relying on PKA. We found that the protective effect of PDE4D siRNA on pAMPK was blocked by PKI (Supplementary Fig. 6a, b), suggesting that PDE4D negatively regulated AMPK phosphorylation via PKA.

Additionally, AMPK activation inhibits phosphorylation of myosin phosphatase targeting subunit 1 (MYPT1) and myosin light chain (MLC), consequently attenuating SMCs contraction^20^. Therefore, we hypothesized that PDE4D may further increase MYPT1 and MLC phosphorylation by suppressing AMPK activation, promoting Ang II-induced SMCs contraction. Indeed, Ang II increased MYPT1 and MLC phosphorylation, whereas PDE4D siRNA suppressed MYPT1 and MLC phosphorylation (Fig. 4f, h, i). Moreover, the effect of PDE4D siRNA on reducing MYPT1 and MLC phosphorylation was largely reversed by PKI (Supplementary Fig. 6a, c, d). We also used AMPK inhibitor (Compound C, 20 μM, 2 h) for further validation. The inhibitory effect of PDE4D siRNA on pMYPT1 and pMLC was blocked by Compound C (Supplementary Fig. 6e–g). These results suggest that PDE4D promotes SMCs contraction via inhibition of PKA activity and AMPK phosphorylation, and conversely promotes MYPT1 and MLC phosphorylation.

### PDE4D promotes vasocontraction through the PKA-AMPK-MYPT1-MLC signaling pathway in Ang II-induced mice hypertension

**Fig. 5:**
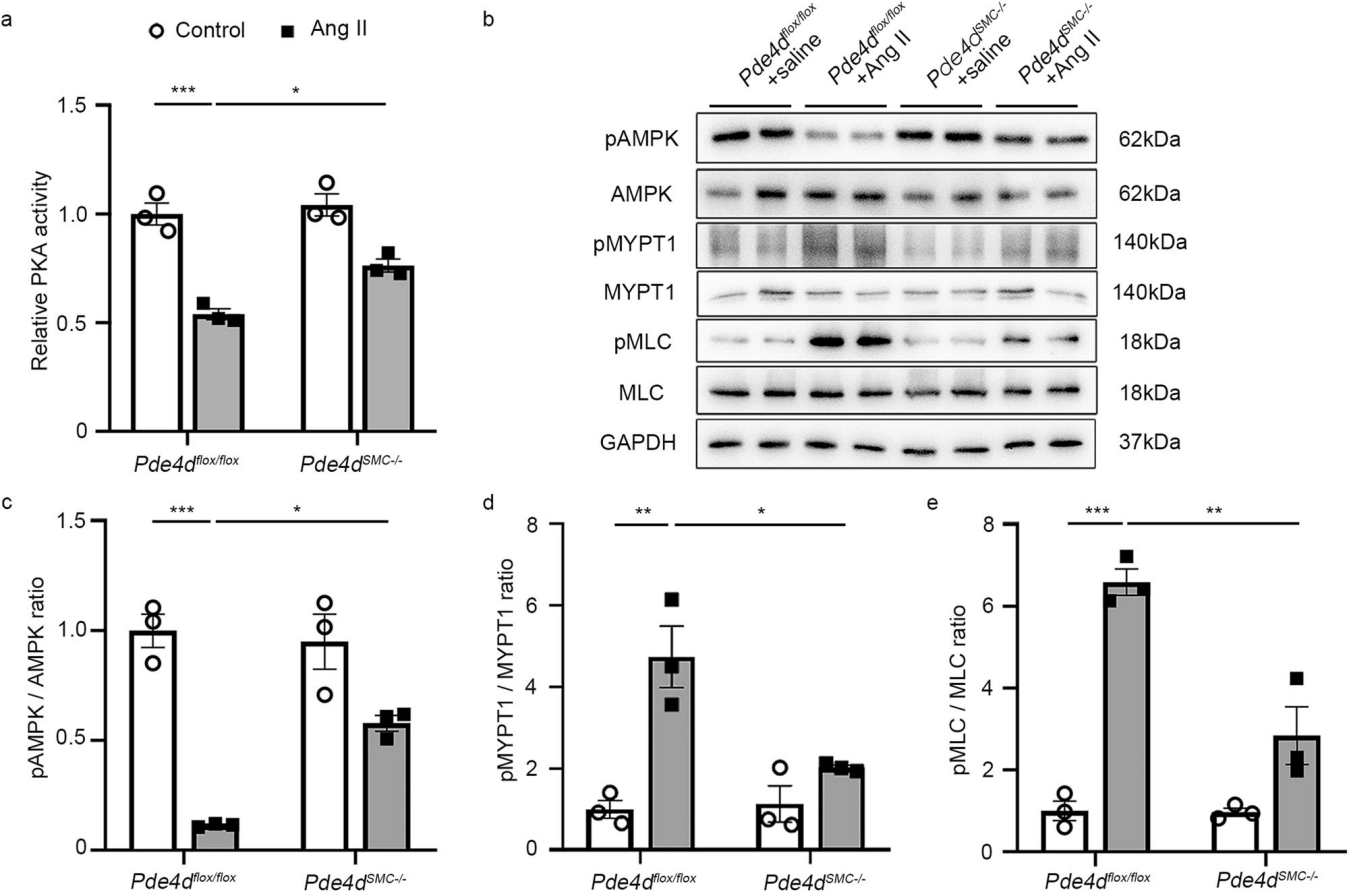
PDE4D promotes vasocontraction through the PKA-AMPK-MYPT1-MLC signaling pathway in vivo. **a** PKA kinase activity in aorta tissues. **b** Representative western blot showing pAMPK, AMPK, pMYPT1, MYPT1, pMLC, and MLC expression in aorta tissues from *Pde4d*^*flox/flox*^ and *Pde4d*^*SMC−/−*^ mice with or without Ang II treatment. **c** Quantification of pAMPK expression normalized to AMPK protein. **d** Quantification of pMYPT1 expression normalized to MYPT1 protein. **e** Quantification of pMLC expression normalized to MLC protein. *n* = 3 per group. Data are expressed as mean ± SEM. Two-way ANOVA with Bonferroni’s post hoc test was performed to compare the difference between the multiple groups. *p < 0.05, **p < 0.01, and ***p < 0.001.

To further validate the mechanism identified in vitro above, we detected PKA activity and AMPK, MYPT1, and MLC phosphorylation in mice aorta tissues. Consistently, Ang II infusion reduced PKA activity in mice aortas, and this reduction was reversed in *Pde4d*^*SMC−/−*^ Ang II mice (Fig. 5a). Western blot also exhibited that Ang II infusion reduced AMPK phosphorylation in the aorta, an effect which was recovered in *Pde4d*^*SMC−/−*^ mice (Fig. 5b, c). Ang II infusion also increased MYPT1 and MLC phosphorylation, and again *Pde4d*^*SMC−/−*^ mice exhibited reduced Ang II-induced MYPT1 and MLC phosphorylation (Fig. 5b, d, e). These results suggest that PDE4D promotes vasocontraction, and thus contributes to Ang II-induced hypertension in mice, through the PKA-AMPK-MYPT1-MLC signaling pathway.

### PDE4 inhibitor rolipram attenuates Ang II-induced hypertension

**Fig. 6:**
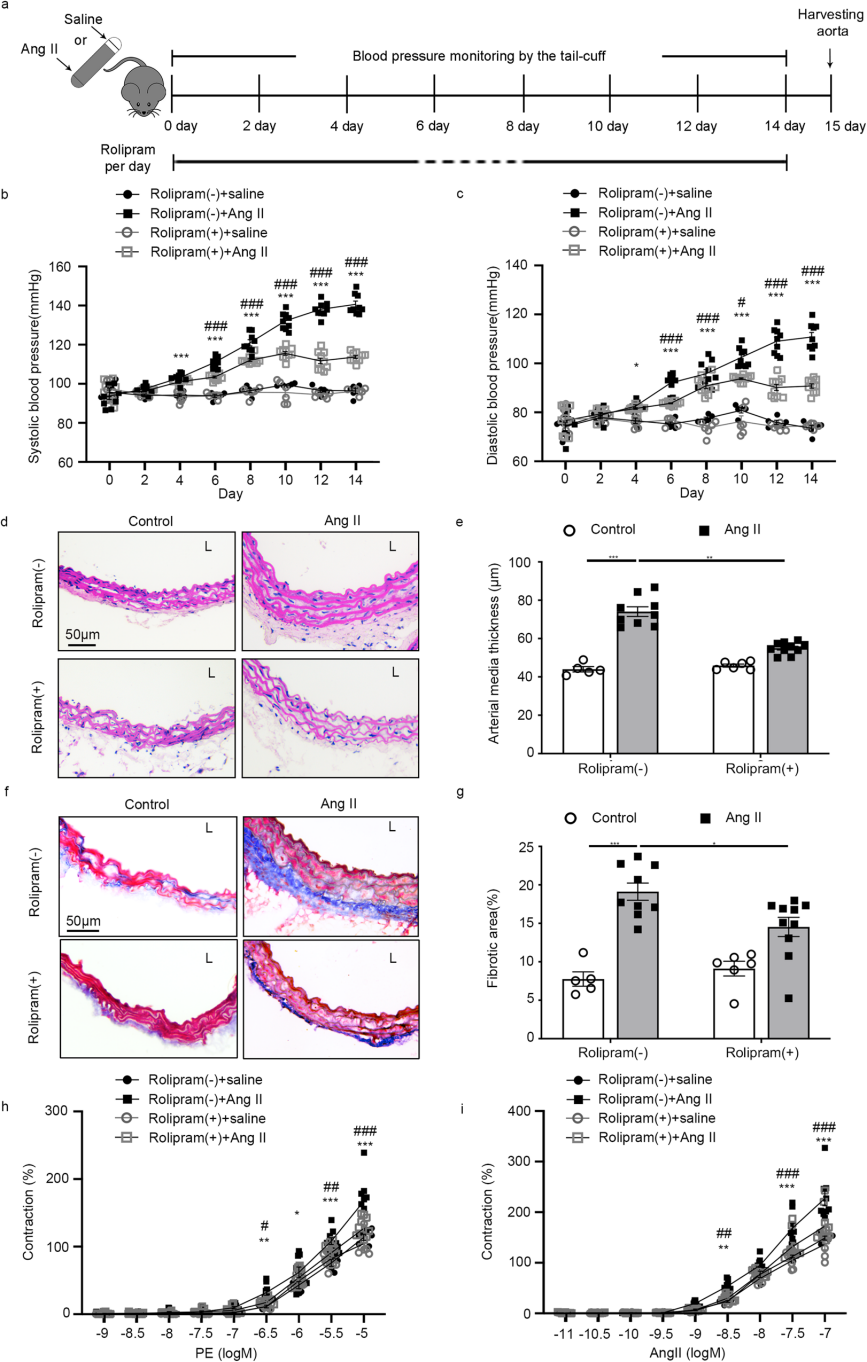
Effect of rolipram on Ang II-induced hypertension in mice. **a** Scheme of hypertensive mice treated with vehicle or rolipram. Rolipram (3 mg kg^−1^ day^−1^) was orally administered daily for 14 days. **b** SBP and **c** DBP were measured in wild-type (WT) mice with or without Ang II/rolipram treatment: *n* = 5 in Rolipram (−) + saline group, *n* = 9 in Rolipram (−) + Ang II group, *n* = 6 in Rolipram (+) + saline group, and *n* = 10 in Rolipram (+) + Ang II group. Data are expressed as mean ± SEM. Two-way ANOVA with Bonferroni’s post hoc test was performed to compare the difference between the multiple groups. *p < 0.05, ***p < 0.001 for Rolipram (−) + Ang II group vs. Rolipram (−) + saline group, and ^#^*p* < 0.05, ^###^*p* < 0.001 for Rolipram (+) + Ang II group vs. Rolipram (−) + Ang II group. **d** Representative H&E staining under the indicated experimental conditions. **e** Measurement of arterial wall media thickness including all mice in **d**. **f** Representative masson-trichrome staining under the indicated experimental conditions. **g** Quantification of the positively stained area to the aortic wall area including all mice in **f**. Data are expressed as mean ± SEM. Two-way ANOVA with Bonferroni’s post hoc test was performed to compare the difference between the multiple groups. *p < 0.05, **p < 0.01, ***p < 0.001 in **e**, **g**. Concentration-response curves for **h** PE and **i** Ang II induced vasocontraction of mesenteric resistance artery in WT mice with or without Ang II/rolipram treatment including all mice. Data are expressed as mean ± SEM. Two-way ANOVA with Bonferroni’s post hoc test was performed to compare the difference between the multiple groups. *p < 0.05, **p < 0.01, ***p < 0.001 for Rolipram (−) + Ang II group vs. Rolipram (−) + saline group group. ^#^*p* < 0.05, ^##^*p* < 0.01, ^###^*p* < 0.001 for Rolipram (+) + Ang II group vs. Rolipram (−) + Ang II group. L lumen.

As known, there are several PDE4 inhibitors used in clinical therapy, for example, rolipram as the first generation of PDE4 inhibitor for neuroinflammation^31^. However, there is no specific inhibitor for PDE4D in pre-clinical experiment. Then, we tried to determine the pharmacological effect of PDE4 inhibitor rolipram on hypertension. We treated WT male mice at 8 weeks age with rolipram (3 mg kg^−1^ day^−1^) or vehicle daily via gavage for 14 days (Fig. 6a). Notably, rolipram reduced Ang II-induced increase of BP [Rolipram (−) + Ang II group vs. Rolipram (+) + Ang II group; SBP: 140.67 ± 1.69 mmHg vs. 113.74 ± 0.80 mmHg, 19.14% reduced by Rolipram (+) + Ang II group; DBP: 110.76 ± 1.79 mmHg vs. 90.76 ± 0.87 mmHg, 18.06% reduced by Rolipram (+) + Ang II group; Fig. 6b, c]. Besides, Ang II-induced vessel wall media thickening was inhibited by rolipram accroding to H&E staining (Fig. 6d, e). In addition, masson-trichrome staining demonstrated that rolipram reversed Ang II-induced vascular fibrosis (Fig. 6f, g). To further confirm the effect of rolipram on vasocontraction, we detected the ex vivo vascular function by using PE and Ang II to induce mesenteric arterioles contraction. We found that rolipram also suppressed vasocontraction observably [Rolipram (−) + Ang II group vs. Rolipram (+) + Ang II group; 10^−5^ M PE: 167.04% ± 11.79% vs. 126.79% ± 5.31%, 24.1% reduced by Rolipram (+) + Ang II group; 10^−7^ M Ang II: 227.06% ± 14.38% vs. 172.43% ± 10.56%, 23.62% reduced by Rolipram (+) + Ang II group; Fig. 6h, i]. These results indicate a pharmacological impact of rolipram in preventing hypertension in mice.

### Rolipram attenuates Ang II-induced hypertension through the inhibition of SMC PDE4D

**Fig. 7:**
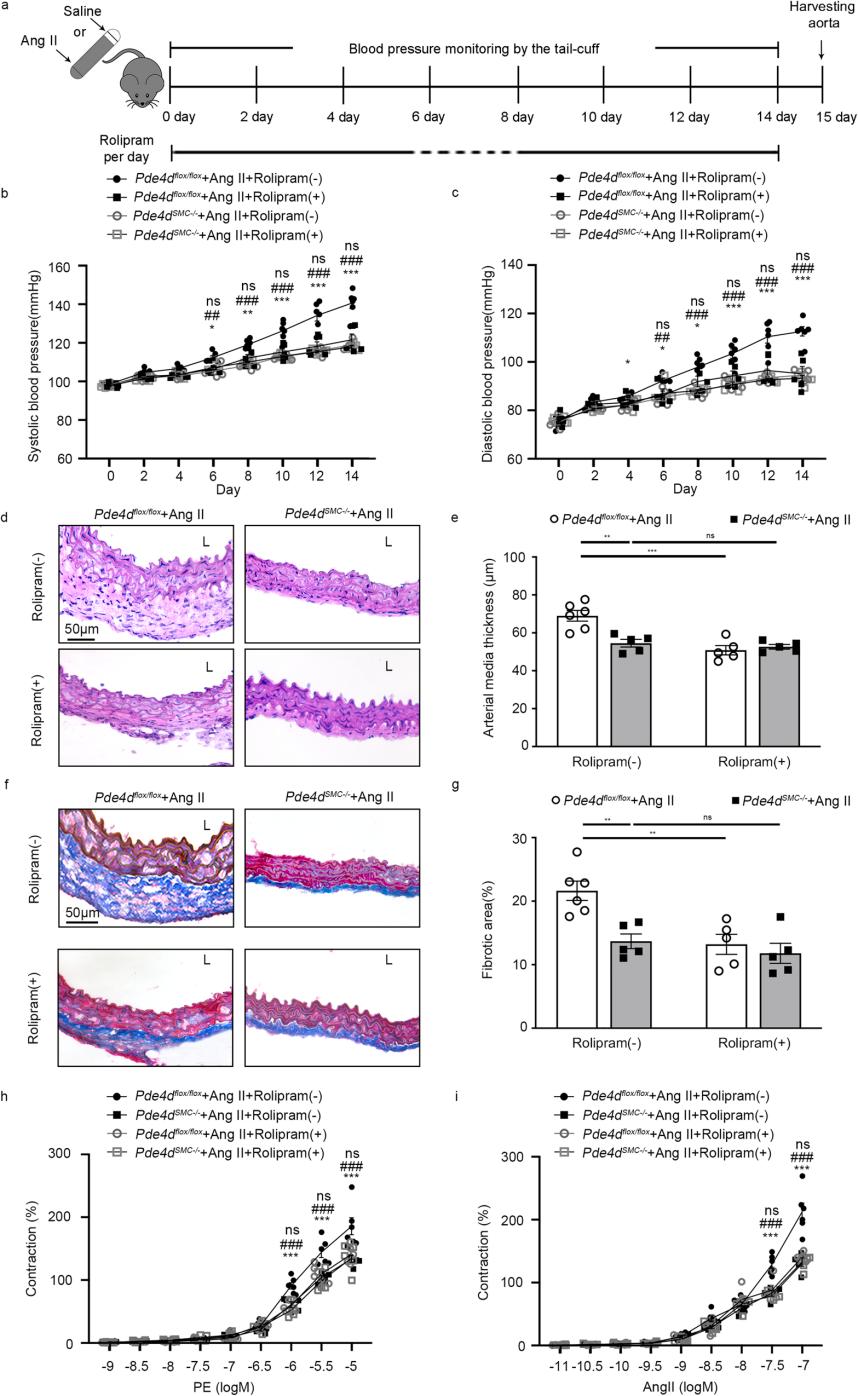
Rolipram attenuates Ang II-induced hypertension through the inhibition of SMCs PDE4D. **a** Scheme of *Pde4d*^*flox/flox*^ and *Pde4d*^*SMC−/−*^ mice infused with Ang II and/or treated with rolipram. Rolipram (3 mg kg^−1^ day^−1^) was orally administered daily for 14 days. **b** SBP and **c** DBP were measured in *Pde4d*^*flox/flox*^ and *Pde4d*^*SMC−/−*^ mice infused with Ang II and/or rolipram treatment: *n* = 6 in *Pde4d*^*flox/flox*^ + Ang II + Rolipram (−) group, *n* = 5 in *Pde4d*^*flox/flox*^ + Ang II + Rolipram (+) group, *n* = 5 in *Pde4d*^*SMC−/−*^ + Ang II + Rolipram (−) group, and *n* = 5 in *Pde4d*^*SMC−/−*^ + Ang II + Rolipram (+) group. Data are expressed as mean ± SEM. Two-way ANOVA with Bonferroni’s post hoc test was performed to compare the difference between the multiple groups. *p < 0.05, **p < 0.01, ***p < 0.001 for *Pde4d*^*flox/flox*^ + Ang II + Rolipram (−) group vs. *Pde4d*^*flox/flox*^ + Ang II + Rolipram (+) group, ^##^*p* < 0.01, ^###^*p* < 0.001 for *Pde4d*^*flox/flox*^ + Ang II + Rolipram (−) group vs. *Pde4d*^*SMC−/−*^ + Ang II + Rolipram (−) group, and ns no significant difference for *Pde4d*^*SMC−/−*^ + Ang II + Rolipram (−) group vs. *Pde4d*^*SMC−/−*^ + Ang II + Rolipram (+) group. **d**, **e** Representative H&E staining and measurement of arterial wall media thickness including all mice. **f**, **g** Representative masson-trichrome staining and quantification of the positively stained area to the aortic wall area including all mice. Data are expressed as mean ± SEM. Two-way ANOVA with Bonferroni’s post hoc test was performed to compare the difference between the multiple groups. **p < 0.01, ***p < 0.001, and ns no significant difference in **e**, **g**. Concentration-response curves for **h** PE and **i** Ang II induced vasocontraction of mesenteric resistance artery. Data are expressed as mean ± SEM. Two-way ANOVA with Bonferroni’s post hoc test was performed to compare the difference between the multiple groups. ***p < 0.001 for *Pde4d*^*flox/flox*^ + Ang II + Rolipram (−) group vs. *Pde4d*^*flox/flox*^ + Ang II + Rolipram (+) group, ^###^*p* < 0.001 for *Pde4d*^*flox/flox*^ + Ang II + Rolipram (−) group vs. *Pde4d*^*SMC−/−*^ + Ang II + Rolipram (−) group, and ns no significant difference for *Pde4d*^*SMC−/−*^ + Ang II + Rolipram (−) group vs. *Pde4d*^*SMC−/−*^ + Ang II + Rolipram (+) group. L lumen.

Rolipram has pan inhibitory effects on PDE4A-D, and the inhibition of PDE4 subfamily by rolipram was not tissue or cell specific. To further explore whether the therapeutic effect of rolipram mainly through SMC PDE4D, we established the hypertensive model in *Pde4d*^*flox/flox*^ and *Pde4d*^*SMC−/−*^ mice, and gavaged mice with rolipram (3 mg kg^−1^ day^−1^) or vehicle (Fig. 7a). Consistently with Figs. 2 and 6, SMC *Pde4d* deficiency or rolipram inhibited Ang II-induced increase of both SBP and DBP (Fig. 7b, c). However, there was no difference between *Pde4d*^*SMC−/−*^ + Ang II + Rolipram (−) group and *Pde4d*^*SMC−/−*^ + Ang II + Rolipram (+) group (SBP: 119.0 ± 1.09 mmHg vs. 117.9 ± 0.81 mmHg; DBP: 94.60 ± 0.93 mmHg vs. 93.20 ± 1.14 mmHg; Fig. 7b, c). H&E staining revealed that SMC *Pde4d* deficiency, rolipram or *Pde4d*^*SMC−/−*^ mice with rolipram inhibited Ang II-induced vessel wall media thickening, but there was no change among the three groups indicated above (Fig. 7d, e). Besides, masson-trichrome staining demonstrated that Ang II-induced vascular fibrosis was reversed by SMC *Pde4d* deficiency, rolipram or *Pde4d*^*SMC−/−*^ mice with Rolipram with no changes among the three groups (Fig. 7f, g). Furthermore, mesenteric arterioles contraction caused by PE and Ang II was markedly suppressed by SMC *Pde4d* deficiency or rolipram, meanwhile, there was no change between *Pde4d*^*SMC−/−*^ + Ang II + Rolipram (−) group and *Pde4d*^*SMC−/−*^ + Ang II + Rolipram (+) group (*Pde4d*^*SMC−/−*^ + Ang II + Rolipram (−) group vs. *Pde4d*^*SMC−/−*^ + Ang II + Rolipram (+) group; 10^−5^ M PE: 141.5% ± 6.57% vs. 135.2% ± 9.89%; 10^−7^ M Ang II: 140.5% ± 2.97% vs. 130.4% ± 4.85%; Fig. 7h, i). These results confirmed that rolipram exerts its therapeutic effect on vascular remodeling, vasoconstriction and hypertension mainly by inhibiting PDE4D in SMCs.

## Discussion

**Fig. 8:**
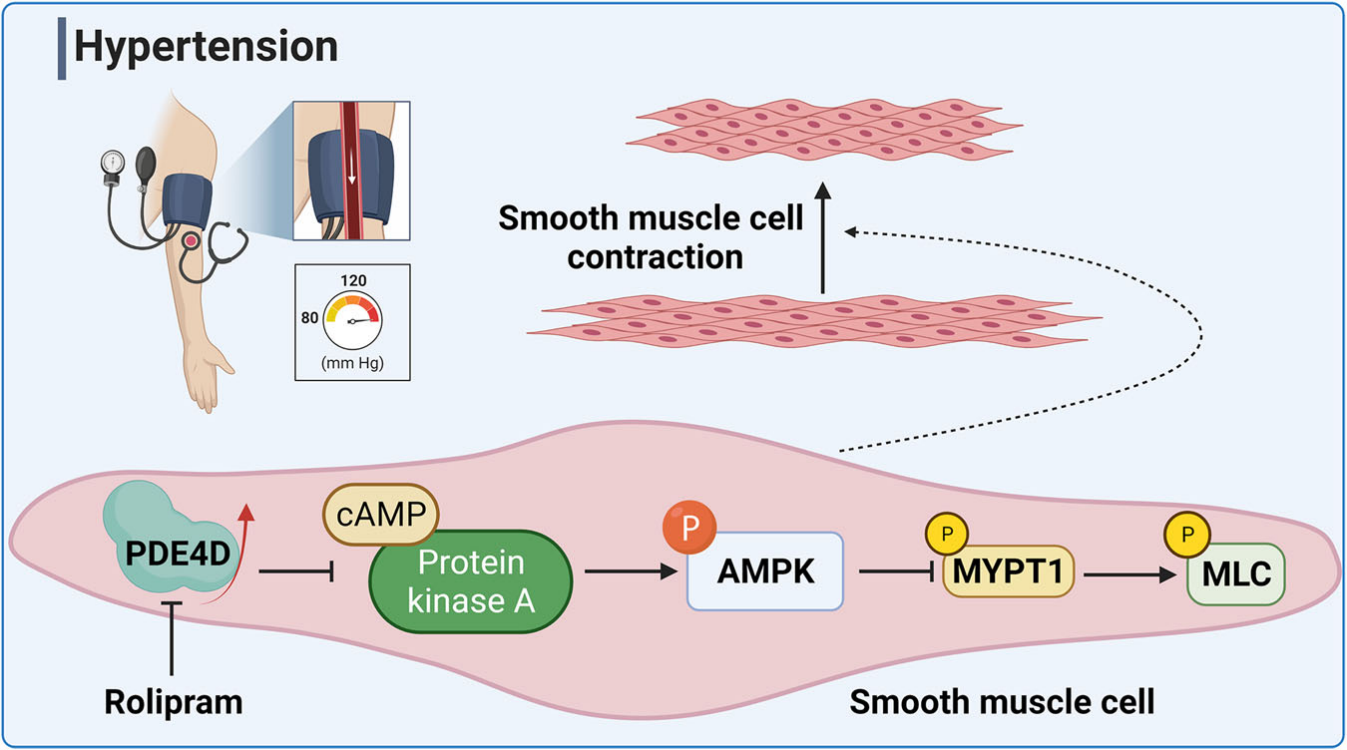
PDE4D promotes Ang II-induced hypertension in mice. PDE4D exacerbates Ang II-induced vasocontraction by affecting SMC contraction, consequently contributing to hypertension. The mechanism by which PDE4D aggravates SMC contraction likely involves the PKA-AMPK-MYPT1-MLC signaling pathway. PDE4 inhibitor, rolipram, showed a therapeutic effect on Ang II-induced hypertension in mice primarily through PDE4D in SMCs. The image was created with BioRender.com, and acquired the permission for use and proper accreditation.

In this study, we observed upregulated PDE4D expression in hypertensive mice aortas, which showed that PDE4D contributes to hypertension. Furthermore, via EC- and SMC-specific *Pde4d* knockout hypertensive mice, these models revealed a causal association between SMC *Pde4d* and vasocontraction in hypertension. To further elucidate this association, we investigated a potential mechanism for PDE4D involvement in SMC contraction and hypertension development, and identified the PKA-AMPK-MYPT1-MLC signaling pathway to be a likely candidate. Importantly, we demonstrated that rolipram, a pan PDE4 inhibitor, relieved Ang II-induced hypertension mainly by inhibiting PDE4D in SMCs, which suggested that PDE4D might represent a potential therapeutic hypertension target (Fig. 8).

Hypertension is well known to be a complex syndrome involving multiple organs, tissues, and cells^32,33^. Among the cell types associated with hypertension, PDE4D is also expressed in fibroblasts^34,35^. Adventitial fibroblasts, another major component of blood vessels, are the primary cause of collagen deposition and aortic stiffening in hypertension^36^. In this study, we observed vascular collagen deposition in Ang II infusion mice. Accordingly, it should not be discounted that PDE4D could further contribute to the development of hypertension by interfering with collagen production in fibroblasts, a possibility warranting future investigation.

Hypertension is commonly associated with inflammation^37,38^, and the cell types involved in inflammation (T lymphocytes^39^, B lymphocytes^40,41^, dendritic cells, monocytes, and macrophages^42^) are all known to promote hypertension. PDE4D has been shown to interact with cytokines, regulate the function of inflammatory cells, and aggravate the inflammatory response^43–45^. While PDE4D’s role in the inflammation response is outside the scope of this study, our findings, along with the body of literature evidence, suggest that PDE4D could also contribute to hypertension via inflammation regulation. Although, further study would be needed to validate this supposition.

While collagen deposition and inflammation may be potential additional mechanisms, we demonstrated a link between PDE4D and the PKA-AMPK signaling pathway. cAMP is known to be involved in signal transduction through PKA regulation^46^. Recently, researchers have found that PKA phosphorylates AMPKα at Thr-172 through the widely expressed tumor suppressor liver kinase B1, ultimately activating AMPK^19,30^. AMPK activity has been linked to numerous cardiovascular diseases, including hypertension, atherosclerosis, and heart failure^20,47–49^. Crucially, AMPK activation lowers BP and suppresses SMC contractility by inhibiting the MYPT1-MLC signaling pathway^20^. Consistent with previous reports, our results exhibited that PDE4D upregulated MYPT1 and MLC phosphorylation by inhibiting the PKA-AMPK signaling pathway, inducing SMCs contraction and thereby, hypertension.

In conclusion, our study provided that PDE4D in SMCs aggravated Ang II-induced hypertension. We identified the mechanism by which PDE4D affected SMCs contraction via in vitro and in vivo experimental models and verified those results through several molecular biology approaches. In addition, rolipram alleviated hypertension mainly through PDE4D in SMCs. This study elucidated PDE4D as a potential target for the treatment of hypertension and, potentially, other cardiovascular diseases.

## Materials and methods

### Animal models

All animal protocols were reviewed and approved by the Ethics Committee of Peking Union Medical College. *Pde4d*-floxed (flanked by LoxP) mice (*Pde4d*^*flox/flox*^), *Tagln*-Cre mice, and *Tek*-Cre mice were generated and obtained by Shanghai Model Organisms Center, Inc. (Shanghai, China). To generate SMC-specific knockout mice (*Pde4d*^*SMC−/−*^) or EC-specific knockout mice (*Pde4d*^*EC−/−*^), *Pde4d*^*flox/flox*^ was crossed with *Tagln*-Cre mice or *Tek*-Cre mice. Genotyping was performed by polymerase chain reaction (PCR) using primers (Supplementary Table 1). *Pde4d*^*flox/flox*^, *Pde4d*^*SMC−/−*^, and *Pde4d*^*EC−/−*^ littermates were used in this research. WT mice (C57BL/6, N11) were obtained from The Jackson Laboratory (Bar Harbor, ME). All mice were housed in temperature-controlled rooms under a 12-h light-dark cycle with water and food ad libitum.

To induce hypertension in *Pde4d*^*flox/flox*^, *Pde4d*^*SMC−/−*^, *Pde4d*^*EC–/–*^, and WT mice, 8-week old male mice were infused with angiotensin II (Ang II, 490 ng kg^−1^ min^–1^; Sigma, Cat#: A9525-50MG) or saline and subcutaneously implanted with osmotic pumps (Alzet MODEL 2002; DURECT, Cupertino, CA) for 14 days^50^. Mice groups: WT mice were divided into two groups randomly, WT mice infused with saline (*n* = 4) or Ang II (*n* = 8); *Pde4d*^*EC−/*−^ mice were divided into four groups randomly, *Pde4d*^*flox/flox*^ mice infused with saline (*n* = 5) or Ang II (*n* = 8), and *Pde4d*^*EC−/−*^ mice infused with saline (*n* = 5) or Ang II (*n* = 8); *Pde4d*^*SMC−/−*^ mice were divided into four groups randomly, *Pde4d*^*flox/flox*^ mice infused with saline (*n* = 5) or Ang II (*n* = 10), and *Pde4d*^*SMC−/−*^ mice infused with saline (*n* = 5) or Ang II (*n* = 8).

To test the effect of rolipram on hypertension, WT, *Pde4d*^*flox/flox*^ and *Pde4d*^*SMC−/−*^ mice at 8 weeks old were infused with Ang II (490 ng kg^−1^ min^−1^) and subcutaneously implanted with osmotic pumps for 14 days. In total, 0.375 mg ml^−1^ rolipram (PDE4 inhibitor, 8 ml kg^−1^ day^−1^, dissolved in ethyl alcohol; Sigma-Aldrich, Cat#: R6520) was administered orally, via gavage, daily for 14 days. WT mice were divided into four groups randomly: Rolipram (−) mice infused with saline (*n* = 5) or Ang II (*n* = 9), and Rolipram (+) mice infused with saline (*n* = 6) or Ang II (*n* = 10). *Pde4d*^*flox/flox*^ and *Pde4d*^*SMC−/−*^ mice were divided into four groups randomly: *Pde4d*^*flox/flox*^ + Ang II mice with Rolipram (−) (*n* = 6) or Rolipram (+) (*n* = 5), and *Pde4d*^*SMC−/−*^ + Ang II mice with Rolipram (−) (*n* = 5) or Rolipram (+) (*n* = 5).

### BP measurement by tail-cuff plethysmography

SBP and DBP in mice were measured using the CODA non-invasive BP system (Kent Scientific Co., Torrington, CT, USA) according to the manufacturer’s instructions^51^. Each mouse was gently placed in a sizeable holder and allowed to acclimate for 5 min. The tail was then threaded through the occlusion cuff and the sensor cuff, which was then attached to the controller. For each measurement, five values of SBP and DBP were recorded for each mouse and their mean values were used as the final result.

### Measurement of mesenteric arteriole tension

Mice were euthanized under pentobarbital sodium (50 mg kg^−^^1^, intraperitoneal). The mesenteric vascular bed was quickly removed, and immersed in Krebs bicarbonate buffer (119 mM NaCl, 25 mM NaHCO_3_, 4.7 mM KCl, 1 mM MgCl_2_, 1.2 mM KH_2_PO_4_, 2.5 mM CaCl_2_, and 11 mM D‐glucose) and gassed with a mixture of 95% O_2_ and 5% CO_2_^52^. Second-order branches of the mesenteric artery (~2 mm long segments), known as the mesenteric arterioles, were suspended with two tungsten wires in the organ chamber containing 5 ml of Krebs buffer solution. The vasocontraction was examined by PE or Ang II. Changes in isometric force were recorded with a Multi Myograph System (610 M, Danish Myo Technology A/S, Aarhus N, Denmark) according to the manufacturer’s instruction. The relative contraction was quantified as the ratio of post stimulation tension to baseline tension.

### Histological and immunohistochemical analysis

Mouse aorta segments were cut at the thoracic aorta, embedded vertically with OCT compound (SAKURA, Cat#:4538), and then stored at −80 °C. Ten to 15 serial frozen sections containing the entire vascular lumen were sectioned using a freezing microtome (Leica CM1860), and then fixed with 4% paraformaldehyde. Frozen sections (6 μm) were stained by immunohistochemical staining of PDE4D (1:100, Abcam, Cat#: ab14613) by the 3-amino-9-ethylcarbazole staining method, H&E (Solarbio, Cat#: G1120), and masson-trichrome staining (Servicebio, Cat#: G1006). Images were photographed using a Leica optical microscope (Leica Microsystems, Germany) and the integrated optical density (IOD) values of positive staining analyzed using Image‐Pro Plus software (Media Cybernetics, USA). For statistical analysis, 5 images per mouse of each group were randomly selected. For PDE4D immunohistochemical staining, the content of PDE4D was quantified as the ratio of positively stained area to the total cross-sectional area of the aortic wall. For masson-trichrome staining, the degree of vascular fibrosis was quantified as the ratio of the positively stained area to the total cross-sectional area of the aortic wall. For H&E staining, arterial wall media thickness was measured using Nikon NIS-Elements image analysis software (Nikon Instruments Inc., Japan).

### Cell culture and small-interfering RNA (siRNA) transfection

RASMCs were purchased from American Type Culture Collection (Manassas, VA, USA) and cultured in SMC medium (ScienCell, Cat#: 1101), containing 5% fetal bovine serum (FBS), 100 U/ml penicillin, and 100 g/ml streptomycin. RASMCs within passage 5 to 12 were used for all experiments. RASMCs were stimulated with 100 nM Ang II (Sigma, Cat#: A9525-50MG) for 24 h before harvest. To knockdown PDE4D, PDE4D siRNA (Ribobio, siB180730051733) and control siRNA (Ribobio, siN0000001-1-5) were purchased from Ribobio (Guangzhou RiboBio Co., Ltd., Guangzhou, China). RASMCs were transfected with 200 nM PDE4D siRNA in 5 μl of Oligofectamine (Invitrogen, Carlsbad, CA, USA, Cat#: 12252011) for 48 h. The siRNA transfection efficiency was determined by real-time polymerase chain reaction (RT-PCR), western blot and immunofluorescence assay (Supplementary Fig. 5a–e).

### Immunofluorescent analysis

RASMCs treated with Ang II (100 nM, 24 h) or PDE4D siRNA (200 nM, 48 h) were fixed with 4% paraformaldehyde. The cells were incubated overnight at 4 °C with primary antibodies against PDE4D (1:100, Abcam, Cat#: ab14613). The antibodies were detected using fluorescein-labeled secondary antibodies (1:1000, Invitrogen, Cat#: A11037). The nuclei were stained with 4′,6-diamidino-2-phenylindole (Abcam, Cat#: ab228549). Images were photographed using a Leica optical microscope (Leica Microsystems, Germany).

### Real-time polymerase chain reaction (RT-PCR)

Total RNA was extracted from mouse aortic tissues or RASMCs using TRIzol reagent (Invitrogen, Carlsbad, CA, Cat#: 15596018) according to the manufacturer’s protocol. Equal quantities of RNA (1000 ng) were reverse transcribed into cDNA (TianGen, KR116-02), and quantitative RT-PCR was performed in a single-color RT-PCR detection system (Bio-Rad, Hercules, CA, USA). The mRNA levels of *Pde4a*, *Pde4b*, *Pde4c*, and *Pde4d* were normalized to the level of the housekeeping gene glyceraldehyde-3-phosphate dehydrogenase (*Gapdh*). *Pde4a*, *Pde4b*, *Pde4c*, and *Pde4d* mRNA expression fold changes compared to one of controls, were calculated using the 2^−ΔΔCt^ method. The RT-PCR primers are shown in Supplementary Table 1.

### Western blot analysis

Protein was extracted from the aortic tissues or RASMCs in a lysis buffer. Equal quantities of protein extract (30 μg per lane) were separated by 8, 10, or 12% SDS–PAGE and transferred to a polyvinylidene fluoride membrane (Merck, Cat#: IPVH00010). The target protein was probed with numerous antibodies: PDE4A (1:1000, Thermo Fisher, Cat#:PA5-115730), PDE4B (1:1000, Cell Signaling Technology, Cat#: 72096S), PDE4C (1:1000, Thermo Fisher, Cat#: PA5-106624), PDE4D (1:1000, Abcam, Cat#: ab171750), AMP-activated protein kinase (AMPK; 1:1000, Cell Signaling Technology, Cat#: 2532S), Phospho-AMPK (1:1000, Cell Signaling Technology, Cat#: 2535S), myosin phosphatase targeting subunit 1 (MYPT1; 1:1000, Cell Signaling Technology, Cat#: 2634S), Phospho-MYPT1 (1:500, Cell Signaling Technology, Cat#: 5163S), MLC (1:1000, Cell Signaling Technology, Cat#: 8505S), and Phospho-MLC (1:1000, Cell Signaling Technology, Cat#: 3675S), respectively. Immunoblotting of the housekeeping protein GAPDH (1:5000, Proteintech, Cat#: 60004-1-Ig) was performed to ensure equal protein loading. Immunoreactive bands were visualized with SuperSignal™ West Pico PLUS Chemiluminescent Substrate (Pierce, Cat#: 34577). The protein expression was measured by analyzing the relative protein band intensity with Image-Pro Plus 6.0 software.

### Protein kinase A (PKA) kinase activity assay

PKA kinase activity in aortic tissues or RASMCs was detected via a PKA kinase activity assay kit (Abcam, Cat#: ab139435) according to the manufacturer’s instructions. Absorbance was measured at OD = 450 nm via a multi-mode microplate reader (BioTek Synergy™ HTX, BioTek Instrument, Inc., Winooski, USA). The relative activity of PKA was quantified as the ratio of the active PKA content to the sample total protein content.

### PDE4 activity assay

PDE4 activity in RASMCs was detected via a PDE4 activity assay kit (Abcam, Cat#: ab139460) according to the manufacturer’s instructions. PDE4 activity was inhibited with rolipram (20 uM) during the test. Total PDE activity assay and PDE activity assay after inhibition of PDE4 were performed for each sample, and PDE4 specific activity was calculated by subtracting inhibitory activity from total activity. Absorbance was measured at OD = 620 nm via a multi-mode microplate reader (BioTek Synergy™ HTX, BioTek Instrument, Inc., Winooski, USA). The relative activity of PDE4 was quantified as the ratio of the active PDE4 content to the sample total protein content.

### Cell contraction assay

RASMCs contraction was detected using a Cell Contraction Assay Kit (Cell Biolabs, Inc., San Diego, CA, USA, Cat#: CBA-201) according to the manufacturer’s instructions^53^. RASMCs were treated with siRNA for 48 h with or without Ang II for 24 h, and then cultured in collagen gel for 48 h to develop mechanical load. The surface image of the collagen gel was captured via digital camera, and analyzed using Image‐Pro Plus software (Media Cybernetics, USA). The percentage of contraction was the ratio of gel contracted surface area to the dish bottom.

### Statistics and reproducibility

Statistical analysis was performed using GraphPad Prism 8 (GraphPad Software Inc., La Jolla, CA). Data are expressed as means ± standard error of mean. Two-tailed Student’s *t* test was performed to compare differences between two groups from at least three independent experiments. One-way ANOVA or two-way ANOVA with Bonferroni’s post hoc test was performed to compare differences between multiple groups, using at least three independent experiments. *p* value < 0.05 was considered statistically significant.

### Reporting summary

Further information on research design is available in the Nature Research Reporting Summary linked to this article.

## Supplementary information

Supplementary Information

Description of Additional Supplementary Files

Supplementary Data 1

Reporting Summary

## Data Availability

Raw data of genotyping and western blot are provided in Supplementary Fig. 7. Source data underlying the graphs are provided in Supplementary Data 1. Other relevant data are available from the corresponding author upon request.
