# Supplementary material for: Phosphodiesterase 4D promotes angiotensin II-induced hypertension in mice via smooth muscle cell contraction
Source: Commun Biol. 2022 Jan 20;5:81. doi: 10.1038/s42003-022-03029-0 (PMC8776755; doi:10.1038/s42003-022-03029-0)
Supplement: Supplementary file 1 — Supplementary Information [file 42003_2022_3029_MOESM1_ESM.pdf]

Phosphodiesterase 4D promotes angiotensin II-induced hypertension in mice via smooth muscle cell contraction

Tianfei Fan[1*], Yangfeng Hou[1*], Weipeng Ge[1], Tianhui Fan[1], Xiaohang Feng[1], Wenjun Guo[1], Xiaomin Song[1], Ran Gao[1#], Jing Wang[1#]

1 State Key Laboratory of Medical Molecular Biology, Institute of Basic Medical Sciences, Chinese Academy of Medical Sciences; Department of Pathophysiology, School of Basic Medicine, Peking Union Medical College, Beijing 100005, China.

# Co-corresponding author:
Dr Jing Wang, State Key Laboratory of Medical Molecular Biology, Institute of Basic Medical Sciences, Chinese Academy of Medical Sciences; Department of Pathophysiology, School of Basic Medicine, Peking Union Medical College, Beijing 100005, China. E-mail: wangjing@ibms.pumc.edu.cn.
Dr Ran Gao, State Key Laboratory of Medical Molecular Biology, Institute of Basic Medical Sciences, Chinese Academy of Medical Sciences; Department of Pathophysiology, School of Basic Medicine, Peking Union Medical College, Beijing 100005, China. E-mail: ggrr1991@outlook.com.
* These authors contributed equally.

Supplementary Figures

Supplementary Figure 1. Blood pressure (BP) of angiotensin (Ang) II induced hypertensive mouse model.

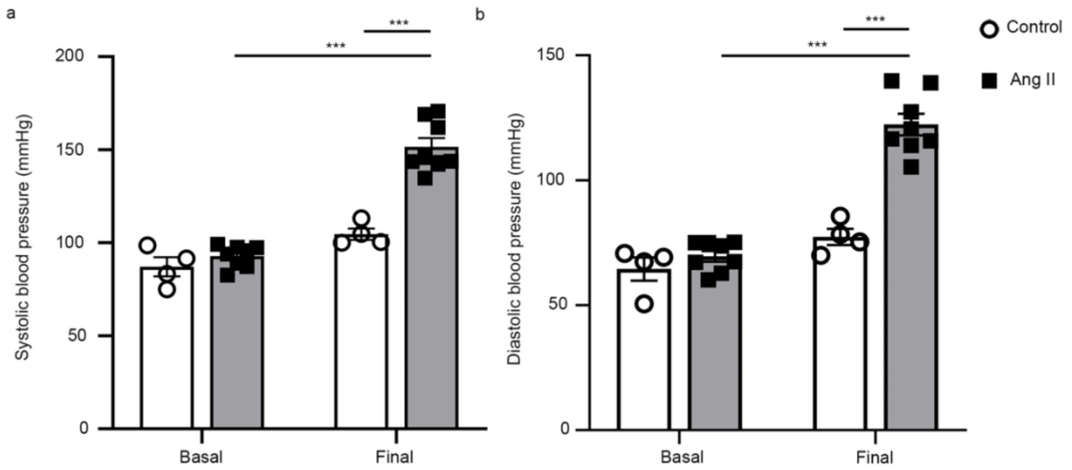

a Systolic and **b** diastolic BPs were measured at baseline and after Ang II infusion for 14 days. Hypertensive mice were induced by Ang II infusion (490 ng/kg/min) for 14 days; controls were infused with saline (n = 4 in control group, n = 8 in Ang II group). Data are expressed as mean ± SEM. Two-way ANOVA with Bonferroni's post hoc test was performed to compare the difference between the multiple groups. ***$P < 0.001$.

Supplementary Figure 2. The expression of Phosphodiesterase (PDE) 4 other isoforms in hypertensive mice.

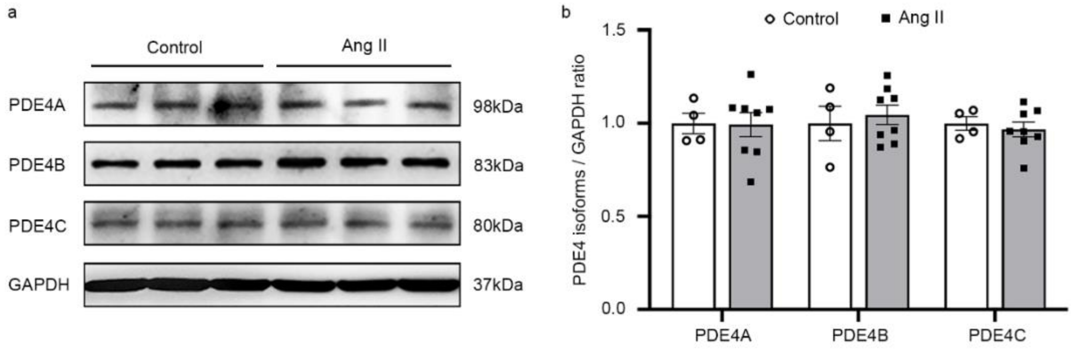

a Representative western blot showing PDE4A, PDE4B and PDE4C expression in aorta tissues. **b** Quantification of PDE4A, PDE4B and PDE4C expression normalized to glyceraldehyde-3-phosphate dehydrogenase (GAPDH) protein (fold change vs. controls respectively).

Supplementary Figure 3. Identification of endothelial cell (EC)-specific knockout phosphodiesterase (PDE) 4D (*Pde4d*$^{EC-/-}$) mice and smooth muscle cell (SMC)-specific knockout PDE4D (*Pde4d*$^{SMC-/-}$) mice.

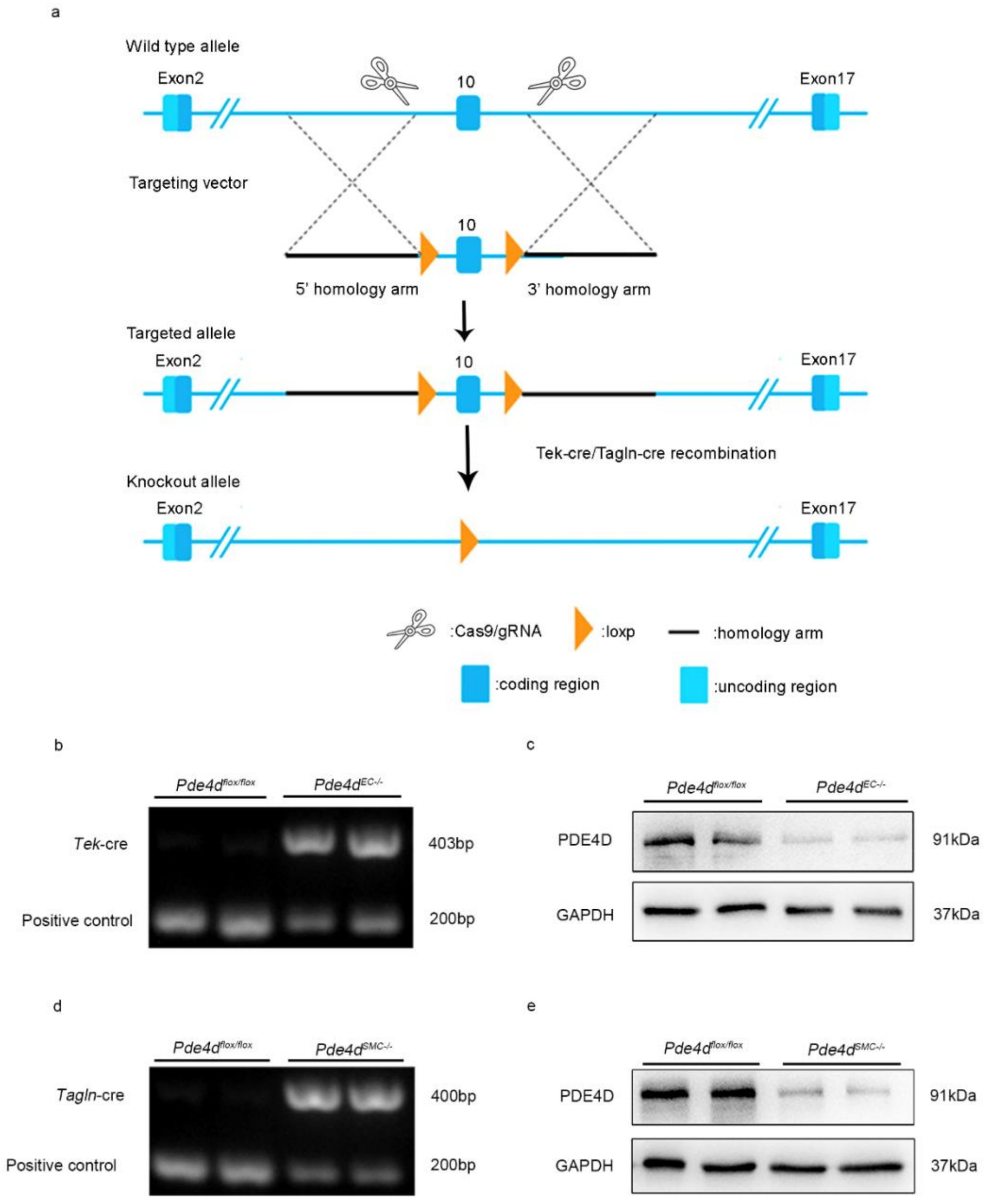

a *Pde4d*$^{EC-/-}$ and *Pde4d*$^{SMC-/-}$ mice were generated via Cre-LoxP recombinase system using CRISPR/Cas9 technology. Exon 10 was deleted via *Tek*-Cre or *Tagln*-Cre–mediated recombination. **b** Genotyping was detected by tail preparation of 2-week old *Pde4d*$^{flox/flox}$ and *Pde4d*$^{EC-/-}$ mice. **c** Representative western blot showing PDE4D expression in the aorta from *Pde4d*$^{flox/flox}$ and *Pde4d*$^{EC-/-}$ mice (n = 2 in each group). **d** Genotyping was detected from the tails of 2-week old *Pde4d*$^{flox/flox}$ and *Pde4d*$^{SMC-/-}$ mice. **e** Representative western blot showing PDE4D expression in the aorta from *Pde4d*$^{flox/flox}$ and *Pde4d*$^{SMC-/-}$ mice (n = 2 in each group).

Supplementary Figure 4. The expression of PDE4 isoforms after stimulated with Ang II in rat aorta smooth muscle cells (RASMCs).

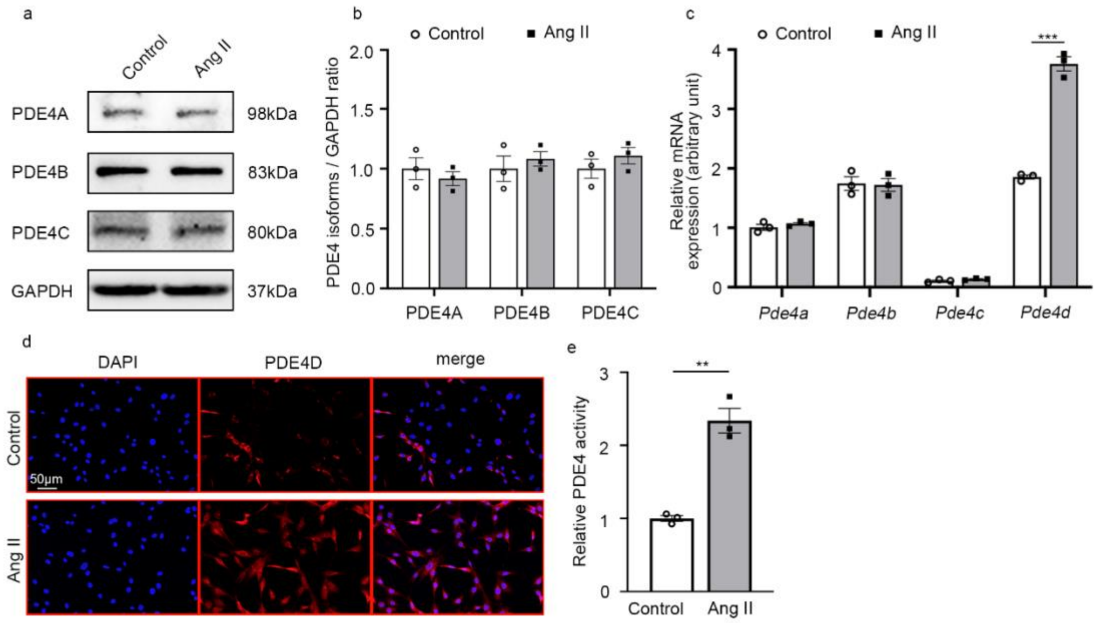

a Representative western blot showing PDE4A, PDE4B and PDE4C expression in RASMCs treated with control or Ang II (100 nM, 24 hrs). **b** Quantification of PDE4A, PDE4B and PDE4C expression normalized to GAPDH protein (fold change vs. controls respectively). **c** Real-time polymerase chain reaction (RT-PCR) to measure *Pde4a*, *Pde4b*, *Pde4c*, and *Pde4d* mRNA expression in RASMCs with control or Ang II (100 nM, 24 hrs) (fold change vs. one of controls). **d** Representative images of immunofluorescence analysis for PDE4D and 4',6-diamidino-2-phenylindole (DAPI, nucleus marker) in RASMCs stimulated with or without Ang II (100 nM, 24hrs). **e** Protein PDE4 activity in RASMCs with or without Ang II. n = 3 per group. Data are expressed as mean ± SEM. Two-tailed Student's t test was performed to compare differences between two groups. $^{**}P < 0.01$, $^{***}P < 0.001$.

Supplementary Figure 5. The expression of PDE4 isoforms after transfected with PDE4D small interfering RNA (siRNA) in RASMCs.

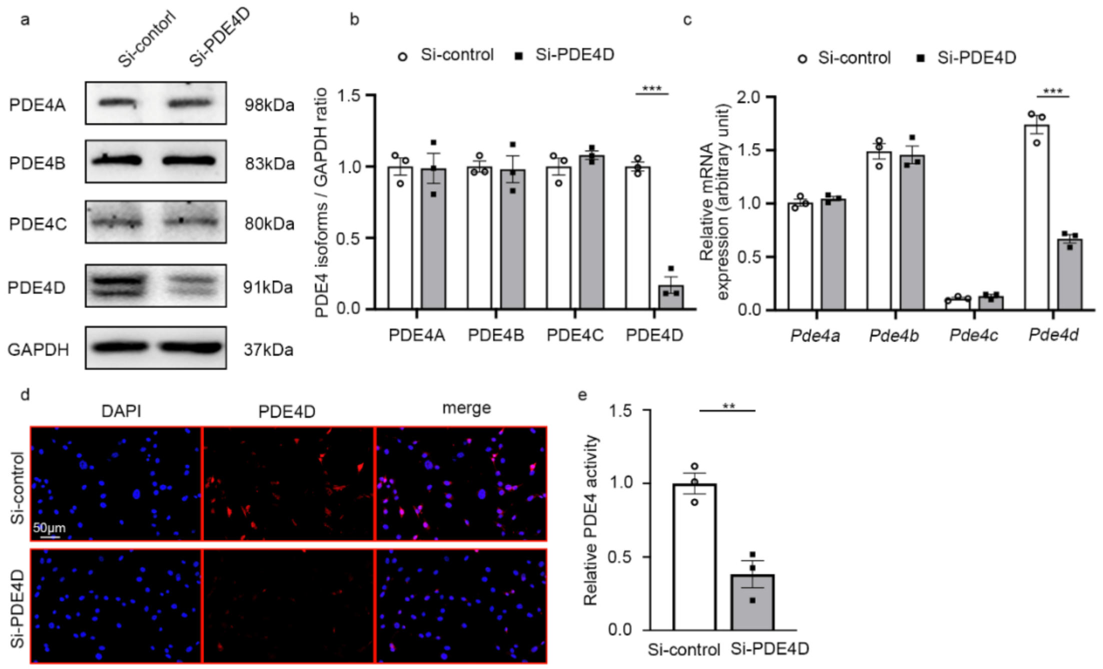

a Representative western blot showing PDE4A, PDE4B, PDE4C and PDE4D expression in RASMCs transfected with control siRNA or PDE4D siRNA (200 nM, 48 hrs). **b** Quantification of PDE4A, PDE4B, PDE4C and PDE4D expression normalized to GAPDH protein (fold change vs. controls respectively). **c** RT-PCR analysis to measure *Pde4a*, *Pde4b*, *Pde4c*, and *Pde4d* mRNA expression in RASMCs with control siRNA or PDE4D siRNA (200 nM, 48 hrs) (fold change vs. one of controls). **d** Representative images of immunofluorescence analysis for PDE4D and DAPI in RASMCs with control siRNA or PDE4D siRNA (200 nM, 48 hrs). **e** Protein PDE4 activity in RASMCs with control siRNA or PDE4D siRNA (200 nM, 48 hrs). n = 3 per group. Data are expressed as mean ± SEM. Two-tailed Student's t test was performed to compare differences between two groups. $**P < 0.01$, $***P < 0.001$.

Supplementary Figure 6. PDE4D antagonizes PKA-AMPK-mediated phosphorylation of MYPT1-MLC signaling pathway *in vitro*.

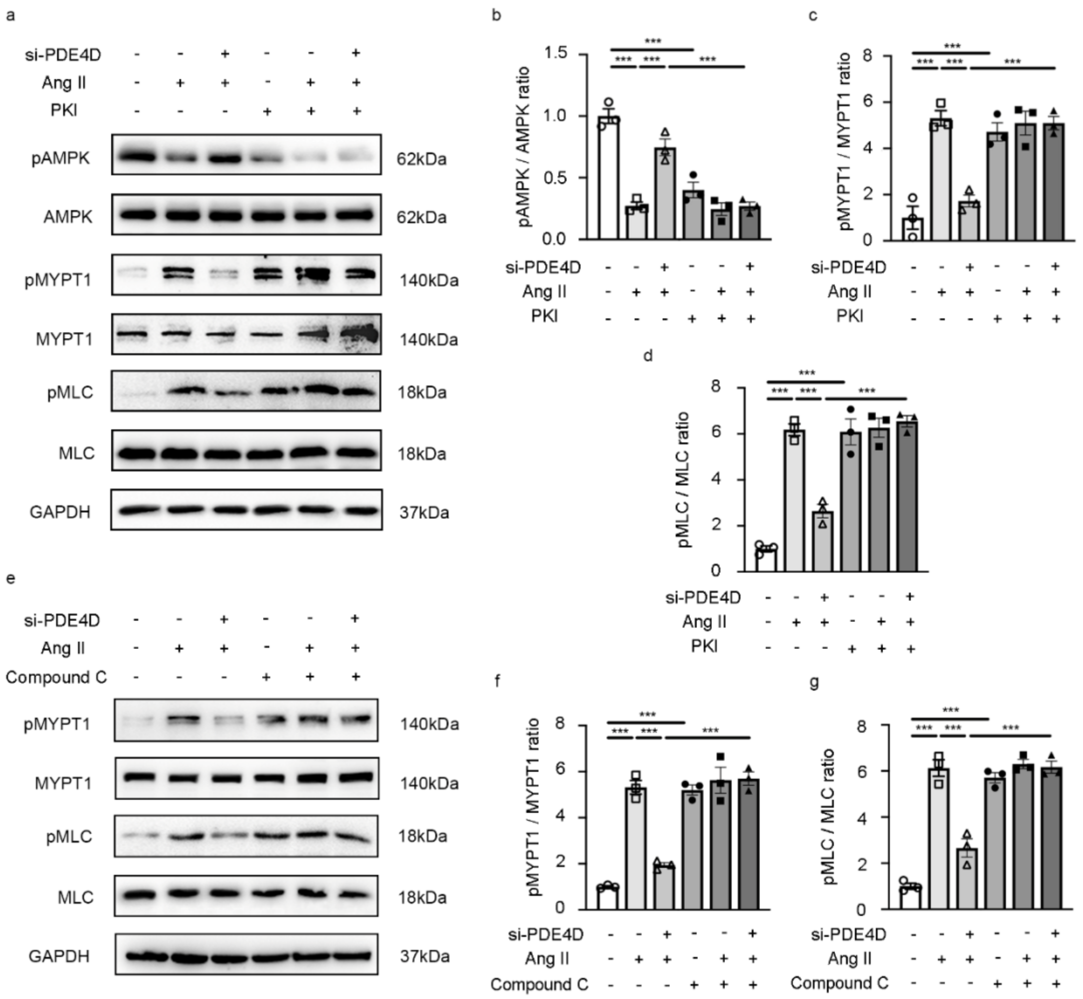

a Representative western blot exhibiting pAMPK, AMPK, pMYPT1, MYPT1, pMLC, and MLC expression in SMCs treated with PKI (PKA inhibitor, 10 µM, 60 min) and / or Ang II (100 nM, 24 hrs) and / or PDE4D siRNA (200 nM, 48 hrs) as indicated. **b** Quantification of pAMPK expression normalized to AMPK protein. **c** Quantification of pMYPT1 expression normalized to MYPT1 protein. **d** Quantification of pMLC expression normalized to MLC protein. **e** Representative western blot exhibiting pMYPT1, MYPT1, pMLC, and MLC expression in SMCs treated with Compound C (AMPK inhibitor, 20 µM, 2 hrs) and / or Ang II (100 nM, 24 hrs) and / or PDE4D siRNA (200 nM, 48 hrs) as indicated. **f** Quantification of pMYPT1 expression normalized to MYPT1 protein. **g** Quantification of pMLC expression normalized to MLC protein. n = 3 per group. Data are expressed as mean ± SEM. One-way ANOVA with Bonferroni's post hoc test was performed to compare the difference between the multiple groups. ***$P < 0.001$.

Supplementary Figure 7. Original images of the genotyping and western blot analysis.

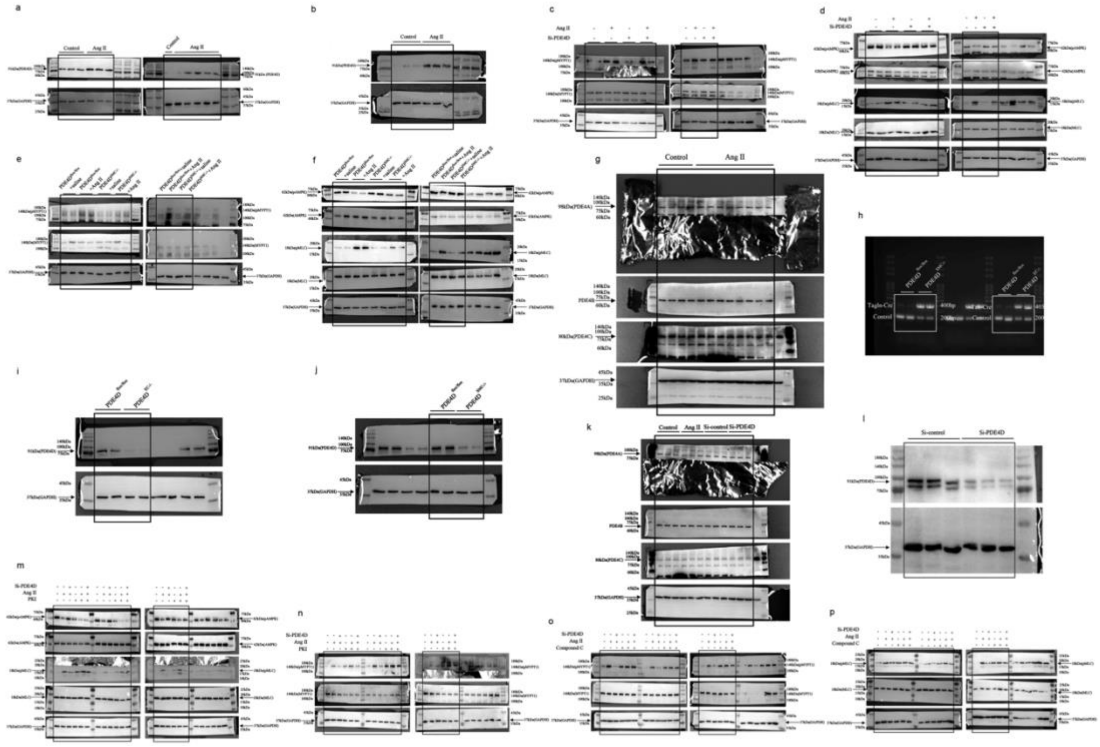

a The original images of western blot analysis in Figure 1b. **b** The original images of western blot analysis in Figure 4a. **c** The original images of western blot analysis in Figure 4f middle panels (pMYPT1 and MYPT1). **d** The original images of western blot analysis in Figure 4f upper and lower panels (pAMPK, AMPK, pMLC and MLC). **e** The original images of western blot analysis in Figure 5b middle panels (pMYPT1 and MYPT1). **f** The original images of western blot analysis in Figure 5b upper and lower panels (pAMPK, AMPK, pMLC and MLC). **g** The original images of western blot analysis in Supplementary Figure 2a. **h** The original images of western blot analysis in Supplementary Figure 3b, d. **i** The original images of western blot analysis in Supplementary Figure 3c. **j** The original images of western blot analysis in Supplementary Figure 3e. **k** The original images of western blot analysis in Supplementary Figure 4a, 5a. **l** The original images of western blot analysis in Supplementary Figure 5a. **m** The original images of western blot analysis in Supplementary Figure 6a middle panels (pMYPT1 and MYPT1). **n** The original images of western blot analysis in Supplementary Figure 6a upper and lower panels (pAMPK, AMPK, pMLC and MLC). **o** The original images of western blot analysis in Supplementary Figure 6e upper panel (pMYPT1 and MYPT1). **p** The original images of western blot analysis in Supplementary Figure 6e lower panel (pMLC and MLC).

Supplementary Table

Supplementary Table 1. Primer sequences

PDE: phosphodiesterase; GAPDH: glyceraldehyde-3-phosphate dehydrogenase; Ko: knockout.

Genes	Species	Forward-Primer	Reverse-Primer
Pde4a	Mouse	AAAGCTGGTACACACCGGAAG	CAGGCCCCATTTGCTCAAGT
Pde4b	Mouse	CAACGCCAGACACTCAGGAA	AGAACACCGGAGCTTGTCAC
Pde4c	Mouse	GGTGTGATCCTGAGACGGTT	TTTCGAGGTCAAAGCTGCTC
Pde4d	Mouse	CTCTCGGAGCAAAAGTGCCT	GATTCGCTTCGCAAGTCTGC
Gapdh	Mouse	AGGTCGGTGTGAACGGATTTG	TGTAGACCATGTAGTTGAGGTCA
Pde4a	Rat	TAACCAATGTGCCCATCCCC	AGGCACCAATCCAGCTCTTC
Pde4b	Rat	TGACAAACCTTCACGGAGCA	CCAGCGTCTCCATTGCTAGT
Pde4c	Rat	TTGACTGAGCGAGTCCGAG	TCGGGATCCGATGGTGAAAA
Pde4d	Rat	CCATGTGCAACCAACCATCC	GGTGAGCTCCCGATTAAGCA
Gapdh	Rat	GGTGCTGAGTATGTCGTGGA	ACTGTGGTCATGAGCCCTTC
Pde4d flox	Mouse	TATTGCCCAGGAAACAGTAACA	GACATCGGCCTTTTCTTCTTCTCC
Pde4d Ko	Mouse	TTGCCCAGGAAACAGTAACAGAAG	AAACAAGCCACCAAACAGCAACAA
Tagln-Cre	Mouse	TCGATGCAACGAGTGATGAG	TCCATGAGTGAACGAACCTG
Tek-Cre	Mouse	TCGATGCAACGAGTGATGAG	TCCATGAGTGAACGAACCTG
Positive control	Mouse	CAAATGTTGCTTGTCTGGTG	GTCAGTCGAGTGCACAGTTT
