# Supplementary material for: Phosphodiesterase 4D promotes angiotensin II-induced hypertension in mice via smooth muscle cell contraction
Source: Commun Biol. 2022 Jan 20;5:81. doi: 10.1038/s42003-022-03029-0 (PMC8776755; doi:10.1038/s42003-022-03029-0)
Supplement: Supplementary file 2 — Description of Additional Supplementary Files [file 42003_2022_3029_MOESM2_ESM.pdf]

Description of Additional Supplementary Files

File name: Supplementary Data 1
Description: Original Data of Figure 1-7 and Supplementary Figure 1-6.
