# Supplementary material for: Phosphodiesterase 4D promotes angiotensin II-induced hypertension in mice via smooth muscle cell contraction
Source: Commun Biol. 2022 Jan 20;5:81. doi: 10.1038/s42003-022-03029-0 (PMC8776755; doi:10.1038/s42003-022-03029-0)
Supplement: Supplementary file 4 — Reporting Summary [file 42003_2022_3029_MOESM4_ESM.pdf]

Corresponding author(s): Jing Wang and Ran Gao

Last updated by author(s): Oct 27, 2021

Reporting Summary

Nature Research wishes to improve the reproducibility of the work that we publish. This form provides structure for consistency and transparency in reporting. For further information on Nature Research policies, see our Editorial Policies and the Editorial Policy Checklist.

Statistics

For all statistical analyses, confirm that the following items are present in the figure legend, table legend, main text, or Methods section.

n/a	Confirmed	
☐	☒	The exact sample size (n) for each experimental group/condition, given as a discrete number and unit of measurement
☐	☒	A statement on whether measurements were taken from distinct samples or whether the same sample was measured repeatedly
☐	☒	The statistical test(s) used AND whether they are one- or two-sided<br>*Only common tests should be described solely by name; describe more complex techniques in the Methods section.*
☒	☐	A description of all covariates tested
☒	☐	A description of any assumptions or corrections, such as tests of normality and adjustment for multiple comparisons
☐	☒	A full description of the statistical parameters including central tendency (e.g. means) or other basic estimates (e.g. regression coefficient) AND variation (e.g. standard deviation) or associated estimates of uncertainty (e.g. confidence intervals)
☒	☐	For null hypothesis testing, the test statistic (e.g. F, t, r) with confidence intervals, effect sizes, degrees of freedom and P value noted<br>*Give P values as exact values whenever suitable.*
☒	☐	For Bayesian analysis, information on the choice of priors and Markov chain Monte Carlo settings
☒	☐	For hierarchical and complex designs, identification of the appropriate level for tests and full reporting of outcomes
☒	☐	Estimates of effect sizes (e.g. Cohen's d, Pearson's r), indicating how they were calculated

Our web collection on statistics for biologists contains articles on many of the points above.

Software and code

Policy information about availability of computer code

Data collection	No software was used.
Data analysis	The data were analyzed with GraphPad Prism (vresion 8.0.1), Image-Pro Plus (vresion 6.0.0.260), Leica Application Suite X software and Nikon NIS-Elements image analysis software.

For manuscripts utilizing custom algorithms or software that are central to the research but not yet described in published literature, software must be made available to editors and reviewers. We strongly encourage code deposition in a community repository (e.g. GitHub). See the Nature Research guidelines for submitting code & software for further information.

Data

Policy information about availability of data

All manuscripts must include a data availability statement. This statement should provide the following information, where applicable:

- Accession codes, unique identifiers, or web links for publicly available datasets
- A list of figures that have associated raw data
- A description of any restrictions on data availability

Raw data of the western blot and genotyping are provided in Supplementary Data 1. Source data underlying the graphs are provided in Supplementary Data 2. Other relevant data are available from the corresponding author upon request.

Field-specific reporting

Please select the one below that is the best fit for your research. If you are not sure, read the appropriate sections before making your selection.

- [x] Life sciences
- [ ] Behavioural & social sciences
- [ ] Ecological, evolutionary & environmental sciences

For a reference copy of the document with all sections, see nature.com/documents/nr-reporting-summary-flat.pdf

Life sciences study design

All studies must disclose on these points even when the disclosure is negative.

Sample size	Sample sizes were determined based on our previous studies.
Data exclusions	No data were excluded.
Replication	The experimental findings were reliably reproduced.
Randomization	Mice with each genotype were randomly divided into two groups. For statistical analysis, 5 images per mouse of each group were randomly selected.
Blinding	No blinding was performed.

Reporting for specific materials, systems and methods

We require information from authors about some types of materials, experimental systems and methods used in many studies. Here, indicate whether each material, system or method listed is relevant to your study. If you are not sure if a list item applies to your research, read the appropriate section before selecting a response.

Materials & experimental systems

n/a	Involved in the study	
	x	Antibodies
	x	Eukaryotic cell lines
x		Palaeontology and archaeology
	x	Animals and other organisms
x		Human research participants
x		Clinical data
x		Dual use research of concern

Methods

n/a	Involved in the study	
x		ChIP-seq
x		Flow cytometry
x		MRI-based neuroimaging

Antibodies

Antibodies used	PDE4A (Thermo Fisher, Cat#:PA5-115730), PDE4B (Cell Signaling Technology, Cat#: 72096S), PDE4C (Thermo Fisher, Cat#: PA5-106624), PDE4D for IHC (Abcam, Cat#: ab14613), PDE4D for WB (Abcam, Cat#: ab171750), AMP-activated protein kinase (AMPK; Cell Signaling Technology, Cat#: 2532S), Phospho-AMPK (Cell Signaling Technology, Cat#: 2535S), myosin phosphatase targeting subunit 1 (MYPT1; Cell Signaling Technology, Cat#: 2634S), Phospho-MYPT1 (Cell Signaling Technology, Cat#: 5163S), myosin light chain (MLC; Cell Signaling Technology, Cat#: 8505S), and Phospho-MLC (Cell Signaling Technology, Cat#: 3675S); GAPDH (Proteintech, Cat#: 60004-1-Ig); HRP-labelled rabbit (Invitrogen, Cat#: A24531) and mouse secondary antibody (Invitrogen, Cat#: 32430).
Validation	All antibodies were reasonably validated in previous studies or by vendors.

Eukaryotic cell lines

Policy information about cell lines

Cell line source(s)	Rat aorta smooth muscle cells were obtained from American Type Culture Collection, VA.
Authentication	Cell line was authenticated by the manufacturer.
Mycoplasma contamination	The cell line was tested negative for mycoplasma.
Commonly misidentified lines (See ICLAC register)	No commonly misidentified cell line was used.

Animals and other organisms

Policy information about studies involving animals; ARRIVE guidelines recommended for reporting animal research

Laboratory animals	Male wild-type mice (C57BL/6) at 8 weeks of age were obtained from The Jackson Laboratory (Bar Harbor, ME).Male PDE4D flox/flox, PDE4D EC-/- and PDE4D SMC-/- mice at 8 weeks of age were obtained from Shanghai Model Organisms Center, Inc.
Wild animals	The study did not involve wild animals.
Field-collected samples	The study did not involve samples collected from the field.
Ethics oversight	All animal protocols were reviewed and approved by the Ethics Committee of Peking Union Medical College.

Note that full information on the approval of the study protocol must also be provided in the manuscript.
